# Tuning Conjugation and Charge Transport in Supramolecular Semiconductors via Hydrogen‐Bonding Position

**DOI:** 10.1002/smsc.70383

**Published:** 2026-08-17

**Authors:** Raúl González‐Núñez, Gabriel Martinez, Kyeong‐Im Hong, Wakana Matsuda, Shu Seki, Pablo Lupiáñez‐Garrido, Amparo Ruiz‐Carretero, Rocío Ponce Ortiz

**Affiliations:** ^1^ Instituto de Ciencia de Materiales de Madrid Consejo Superior de Investigaciones Científicas CSIC Madrid Spain; ^2^ Institute Charles Sadron CNRS UPR22 University of Strasbourg Strasbourg France; ^3^ Department of Molecular Engineering Graduate School of Engineering Kyoto University Kyoto Japan; ^4^ Department of Physical Chemistry University of Málaga Málaga Spain; ^5^ Instituto Universitario de Materiales y Nanotecnología IMANA University of Málaga Málaga Spain

**Keywords:** charge transport, conjugation modulation, hydrogen‐bonding, organic semiconductors, supramolecular assembly

## Abstract

Precise control over conjugation pathways is essential for developing high‐performance organic semiconductors, particularly in hydrogen‐bonded systems where subtle structural variations strongly influence hydrogen bond strength, molecular planarity, supramolecular organization, and charge transport. We demonstrate that the relative positioning of hydrogen‐bonding with respect to a π‐conjugated core enables the modulation of conjugation pathways through controlled intra‐ or intermolecular interactions. A series of diketopyrrolopyrrole small molecules bearing amides at defined distances from the conjugated backbone was designed to selectively favor distinct hydrogen‐bonding types and strengths. Our combined approach, including density functional theory, vibrational and electronic spectroscopies, electrochemistry, and solid‐state characterization, reveals that proximal amide groups favor intramolecular hydrogen‐bonding, disrupting backbone planarity and limiting effective π‐conjugation. In contrast, distal amides promote intermolecular hydrogen‐bonding, sometimes involving DPP carbonyl groups, leading to extended conjugation pathways and enhanced supramolecular organization in the solid state. This modulation of hydrogen‐bond topology and strength results in markedly different charge‐carrier dynamics and transport, evidenced by electrodeless photoconductivity measurements and organic field‐effect transistors. Overall, this work establishes hydrogen‐bond and strength, rather than hydrogen‐bonding alone, as key molecular design parameter to modulate conjugation extension and charge transport in hydrogen‐bonded semiconductors, providing general insights for designing functional supramolecular electronic materials.

## Graphical abstract

The position of hydrogen‐bonding motifs relative to a π‐conjugated core determines whether intra‐ or intermolecular hydrogen‐bonding prevails, modulating molecular planarity, conjugation pathways, supramolecular organization, and charge transport. This work establishes hydrogen‐bond type as a key molecular design principle for high‐performance supramolecular organic semiconductors.

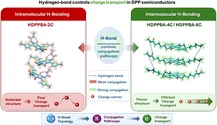

## 1 Introduction

In the rapidly evolving field of organic semiconductors, hydrogen‐bonded materials have emerged as a class of compounds with promising electronic properties and tunable functionalities [1, 2, 3, 4, 5, 6]. Within this broad class of materials, small molecules that integrate π‐conjugated cores with hydrogen‐bonding motifs are of particular interest due to their structural versatility and potential in electronic applications [2, 7, 8]. In these systems, intra‐ and intermolecular hydrogen‐bonding can significantly influence molecular planarity, effective π‐conjugation length, and solid‐state organization, all of which are critical parameters governing charge transport [9, 10, 11, 12]. In this sense, a key design challenge lies in modulating hydrogen‐bonding patterns to favor extended conjugation pathways that facilitate the generation of charge carriers, altering the final electrical performance. In particular, hydrogen‐bonding may involve either pendant functional groups or the conjugated backbone itself, potentially altering conjugation pathways by favoring or suppressing specific electronic couplings. Despite its importance, the role of hydrogen‐bond type, that is, whether hydrogen‐bonding occurs intra‐ or intermolecularly and how it engages the π‐conjugated core, remains insufficiently understood, especially in small‐molecule semiconductors.

A central challenge in the rational design of hydrogen‐bonded semiconductors is therefore not merely the introduction of hydrogen‐bonding motifs, but the controlled modulation of conjugation pathways through precise molecular design. In this context, intramolecular hydrogen‐bonding may rigidify molecular conformations or, conversely, induce backbone distortion, while intermolecular hydrogen‐bonding can promote supramolecular order and electronic delocalization across neighboring molecules. Disentangling these competing effects requires molecular systems in which hydrogen‐bonding type can be systematically tuned without altering the electronic nature of the conjugated core.

Diketopyrrolopyrrole (DPP) derivatives represent an ideal platform to address this challenge. DPP‐based small molecules are well known for their strong light absorption, high charge‐carrier mobilities, and structural robustness, and their electronic properties are highly sensitive to backbone planarity and intermolecular interactions [13, 14]. Recently, hydrogen‐bonding has been successfully exploited in DPP‐based materials to enhance charge transport, improve air stability, and control self‐assembly [3, 5, 6]. Nevertheless, the specific consequences of engaging the DPP carbonyl groups in hydrogen‐bonding, particularly in distinguishing intra‐ versus intermolecular interactions, have only begun to be explored.

In our recent work, we demonstrated that a thiophene‐capped DPP derivative bearing distal amide groups exhibits superior charge transport properties compared to a non hydrogen‐bonding analog [15]. Notably, the enhanced performance was attributed not only to supramolecular aggregation, but to specific intermolecular hydrogen bonding between the DPP carbonyl and a pendant amide of a neighboring molecule. This interaction favored a linear thiophene–DPP–thiophene conjugation pathway while suppressing a competing cross‐conjugated route involving the DPP carbonyls, highlighting hydrogen‐bonding as an effective lever to modulate conjugation pathways [15].

**FIGURE 1:**
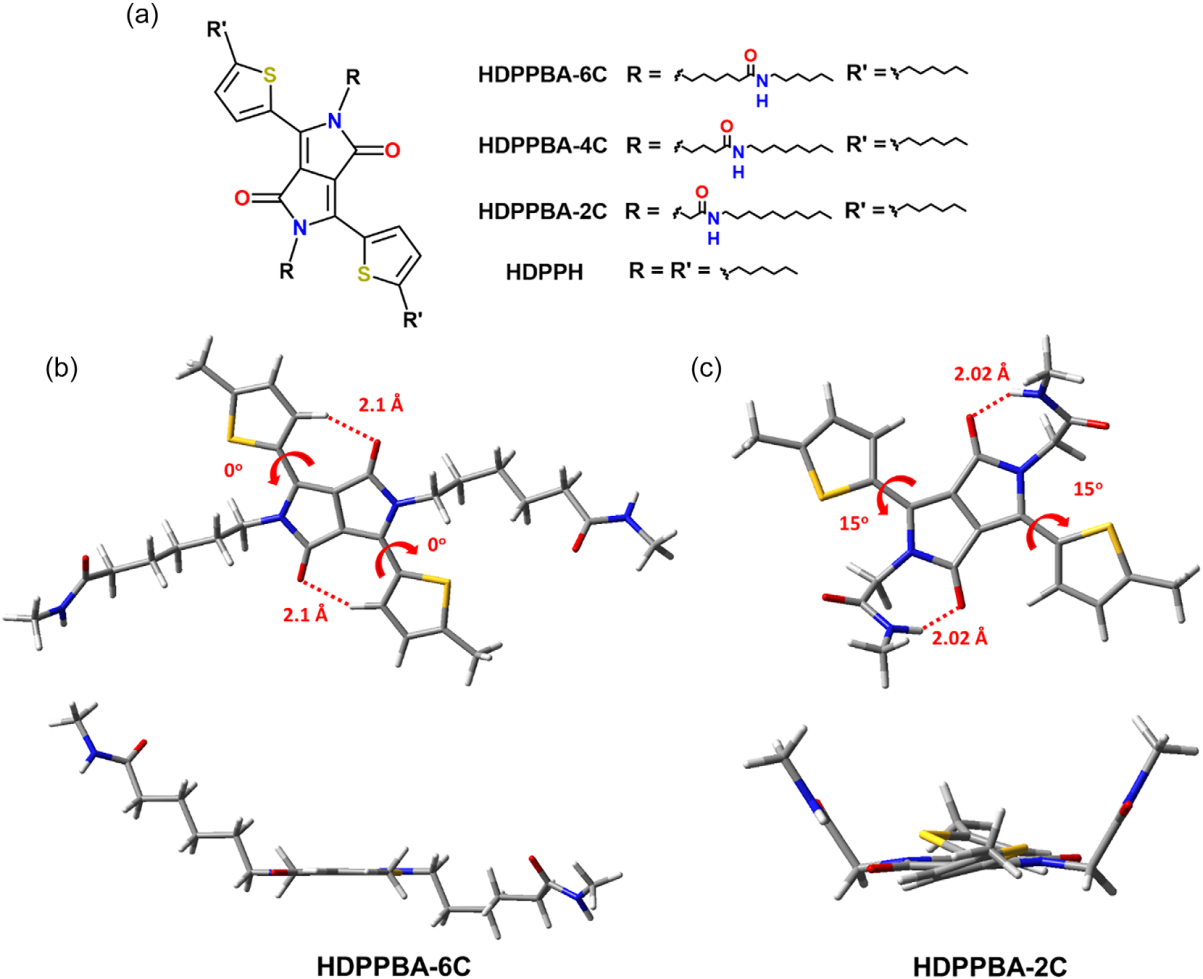
(a) Chemical structures of **HDPPH**, **HDPPBA‐2C**, **HDPPBA‐4C**, and **HDPPBA‐6C**. DFT‐computed global minimum energy geometries for (b) **HDPPBA‐6C** and (c) **HDPPBA‐2C**.

Here, we build on this concept and investigate how the distance between hydrogen‐bonding motifs and the conjugated DPP core governs hydrogen‐bond type, molecular planarity, and electronic properties, while revisiting the effect of the intermolecular hydrogen bonding between the DPP carbonyl and a pendant amide of a neighboring molecule, previously analyzed by us, on molecular structure and conjugation. To this end, we design and synthesize a series of thiophene‐capped DPP small molecules bearing amide functionalities positioned at precisely defined distances from the π‐conjugated backbone, namely **HDPPBA‐2C**, **HPDDBA‐4C**, and **HDPPBA‐6C** (Figure 1a). This molecular design allows selective promotion of either intramolecular or intermolecular hydrogen‐bonding while preserving the electronic identity of the conjugated core. For comparison, we have included as well a control molecule, **HDPPBA** (Figure 1a), which is unable to form hydrogen bonds and was previously synthesized and reported by us [16].

Through a combined experimental and theoretical approach, including density functional theory (DFT) calculations, UV–vis and vibrational spectroscopies, electrochemistry, electrodeless photoconductivity measurements, atomic force microscopy (AFM), and organic field‐effect transistor (OFET) characterization, we demonstrate that hydrogen‐bond type plays a decisive role in the modulation of conjugation pathways and charge transport. Intriguingly, we observe deviations from previously reported conjugation mechanisms, finding a new role for intramolecular hydrogen‐bonding in shaping the electronic landscape of these materials. Proximal amide groups favor intramolecular hydrogen‐bonding that disrupts backbone planarity and limits effective π‐conjugation, whereas distal amide placement promotes intermolecular hydrogen‐bonding, enhanced supramolecular organization, and improved charge transport. Although this study focuses on DPP‐based semiconductors, the underlying design principle, specifically the modulation of conjugation pathways through controlled hydrogen‐bond type, is broadly applicable to other hydrogen‐bonded π‐conjugated small molecules and supramolecular semiconductors.

Together, our results not only hold significance for the development of efficient organic semiconductor materials but also contribute to the broader understanding of the role of hydrogen‐bonding in shaping the electronic properties of functional molecular systems, even those based on particularly small molecules. Ultimately, these findings aim to provide valuable insights that can guide the rational design of hydrogen‐bonded semiconductors, opening new avenues for the progress of organic optoelectronics.

## 2 Results and Discussion

### 2.1 Synthesis

Compounds **1** and **HDPPBA‐6C** were synthesized following a protocol previously reported by our group [16]. On the other hand, **HDPPBA‐2C** and **HDPPBA‐4C** were synthesized from derivative **1** by introducing the hydrogen‐bonding motifs in subsequent steps (Scheme S1) [16]. Initially, product **1** was alkylated using *t*‐butyl bromoacetate and *t*‐butyl 4‐bromobutanoate to afford **HDPP‐2C‐P** and **HDPP‐4C‐P** in 17% and 13% yield, respectively [17]. The low yields are attributed to the reduced solubility of the starting material **1** in acetone, in addition to the expected formation of *N*,*O*‐ and/or *O*,*O’*‐ alkylated isomers as byproducts [18]. Then, a deprotection step was performed using trifluoroacetic acid (TFA) to isolate **HDPP‐2C‐acid** and **HDPP‐4C‐acid** with 78% and 89% yield, respectively. Finally, the resulting diacids were functionalized with 1‐decanamine and 1‐octanamine through a peptide coupling reaction using hexafluorophosphate benzotriazole tetramethyl uronium (HBTU) [19] to obtain **HDPPBA‐2C** and **HDPPBA‐4C** in 79% and 89% yields, respectively (Scheme S1). The final molecules were characterized by ^1^H and ^13^C‐NMR and high‐resolution mass spectrometry (see Supporting Information for detailed description and full characterization).

### 2.2 Structural and Optical Properties

To elucidate how hydrogen‐bond distance influences molecular structure and effective π‐conjugation, ground‐state calculations based on DFT were performed on the amide‐functionalized DPP derivatives. The optimized geometries reveal a pronounced dependence of backbone planarity on the relative position of the amide groups with respect to the conjugated core.

**HDPPBA‐4C** and **HDPPBA‐6C** adopt nearly fully planar conformations, with dihedral angles close to 0° between the DPP core and the flanking thiophene units (Figure 1b). This planarity is stabilized by weak intramolecular O–H interactions between the DPP carbonyl oxygen and the adjacent thiophene hydrogen atoms, which effectively lock the thiophene–DPP–thiophene backbone in a coplanar geometry (Figures 1b and S1 for **HDPPH** and **HDPPBA‐4C**). This preferred conformation between the DPP core and the flanking thiophene rings, predicted by the DFT calculations, is in agreement with several previously reported works on DPP‐based small molecules and polymers [20, 21, 22, 23]. The calculated O–H distance is 2.1 Å, lower than the sum of the van der Waals radii (2.72 Å), corroborating the aforementioned interactions. In contrast, **HDPPBA‐2C** exhibits a nonplanar backbone, with dihedral angles of approximately 15° between the DPP and thiophene units. In this case, the proximity of the amide groups enables the formation of intramolecular hydrogen bonds between the amide N–H and the DPP carbonyl oxygen, suppressing the stabilizing O–H interactions observed in the longer‐spacer derivatives and inducing backbone twisting (Figures 1c and S2). Additional characterization of the proposed intramolecular hydrogen bond in **HDPPBA‐2C** was carried out by variable‐temperature (VT) ^1^H‐NMR spectroscopy in dilute CDCl_3_ solution (0.25 mM). Prior to the VT measurements, concentration‐dependent ^1^H‐NMR experiments were performed over a broad concentration range (20–0.25 μM, Figure S3) for the three H‐bonded derivatives. The amide NH resonance shows only a very limited concentration dependence over this range in the case of **HDPPBA‐2C**, suggesting that intermolecular aggregation is not the dominant contribution under the dilute conditions employed for the VT experiments (Figure S4). Following established procedures used to investigate intramolecular hydrogen‐bonding in supramolecular systems [24, 25], the amide NH resonance exhibits a small upfield shift upon increasing the temperature, whereas the aromatic thiophene protons and nearby aliphatic resonances remain essentially unchanged. This behavior is consistent with a partial weakening of the intramolecular hydrogen bond upon heating rather than changes associated with intermolecular aggregation. Together with the DFT calculations, these observations support the presence of a predominantly intramolecularly hydrogen‐bonded conformer in **HDPPBA‐2C** under dilute, weakly competitive solution conditions.

**FIGURE 2:**
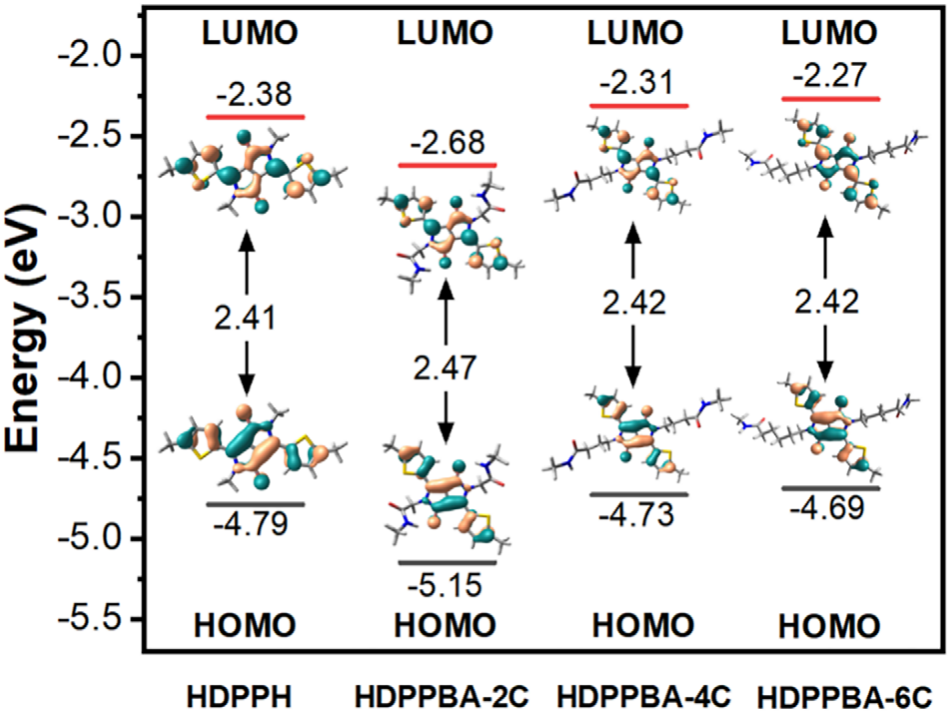
DFT‐calculated molecular energies (B3LYP/6‐31G**) for the studied compounds along with topologies of the HOMO and LUMO orbitals (isovalue of 0.035 a.u).

These structural differences have direct consequences for the electronic structure. The energies and topologies of the studied compounds were also predicted (Figures 2 and S5). As expected, the highest occupied molecular orbital (HOMO) is localized over the entire π‐conjugated core, spanning both the DPP core and the adjacent thiophene rings. On the other hand, the lowest unoccupied molecular orbital (LUMO) is mainly concentrated on the DPP core. DFT calculations point out quite similar HOMO and LUMO energy levels for **HDPPBA‐4C** and **HDPPBA‐6C**, with energy gaps of 2.41−2.42 eV, very similar to those of the control **HDPPH**. On the contrary, in **HDPPBA‐2C**, both HOMO and LUMO energy levels are stabilized with respect to those of **HDPPBA‐4C** and **HDPPBA‐6C**, in around 0.3‐0.41 eV in the case of the LUMO level and 0.36−0.46 for the HOMO level. Thus, the theoretical energy gap is slightly enlarged, being 2.47 eV for **HDPPBA‐2C**. This is ascribed to the planarity loss due to the hydrogen‐bond intramolecular interactions.

Together, the DFT results establish that hydrogen‐bond topology controls molecular planarity and π‐conjugation, providing a structural basis for the distinct electronic and transport properties observed experimentally.

**FIGURE 3:**
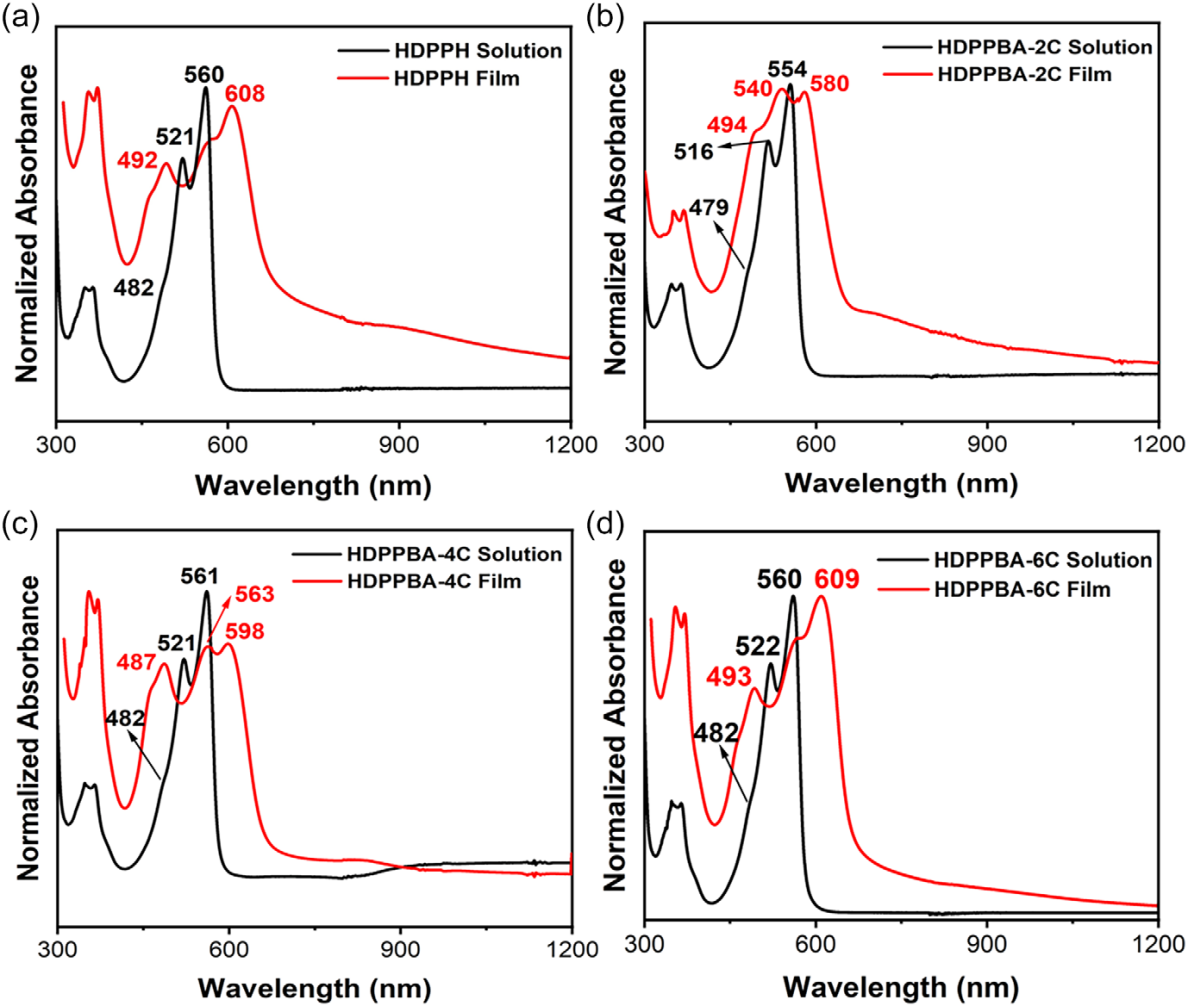
(a) UV–vis spectra in chloroform and as thin film for **HDPPH**, (b) for **HDPPBA‐2C**, (c) for **HDPPBA‐4C**, and (d) for **HDPPBA‐6C**.

The influence of hydrogen‐bond distance from the conjugated core on the optical properties of the DPP derivatives was investigated by UV–vis absorption spectroscopy in solution and in thin films (Figure 3). The results were rationalized with the help of time‐dependent DFT (TD‐DFT) calculations (Figure S6). In dilute chloroform solutions, all three derivatives as well as the control molecule exhibit similar absorption spectra, characterized by two intense bands typical of DPP chromophores, having absorption maxima around *λ* = 520 nm and *λ* = 560 nm (Figure 3a–d, black traces). Although the overall spectra are similar across the series, **HDPPBA‐2C** shows a slight blueshift of the absorption maxima compared to **HDPPBA‐4C** and **HDPPBA‐6C**. This subtle difference is consistent with reduced backbone planarity and shorter effective conjugation length in **HDPPBA‐2C**, in agreement with DFT predictions. The lowest‐energy absorption, assigned to a single‐electron HOMO→LUMO transition (Table S1) [26], appears at comparable wavelengths for the **HDPPH**, **HDPPBA‐4C, and HDPPBA‐6C** compounds, and slightly higher wavelengths for **HDPPBA‐2C** reflecting the limited extent of intermolecular hydrogen‐bonding under these conditions due to good solubility and low aggregation.

In thin films (Figure 3a–d, red traces), the absorption spectra broaden and redshift relative to solution, with main bands around 490 and 610 nm, indicating enhanced intermolecular interactions and solid‐state organization. However, despite the resemblance between the spectra of the three studied molecules, UV–vis absorption spectra already account for the skeleton planarity loss in **HDPPBA‐2C**, since both solution and film UV–vis maxima are slightly blueshifted with respect those of **HDPPH**, **HDPPBA‐4C**, and **HDPPBA‐6C**, with this shift being more accentuated in thin film spectra. These results indicate that differences in hydrogen‐bond type primarily manifest in the solid state, where molecular packing and supramolecular organization amplify their electronic consequences. Note, however, that the redshift in the solid state in **HDPPBA‐6C** is more pronounced than in **HDPPBA‐4C**, indicating also some changes in supramolecular interactions when the amide group is further away from the conjugated skeleton.

### 2.3 Vibrational Spectroscopic Analysis

**FIGURE 4:**
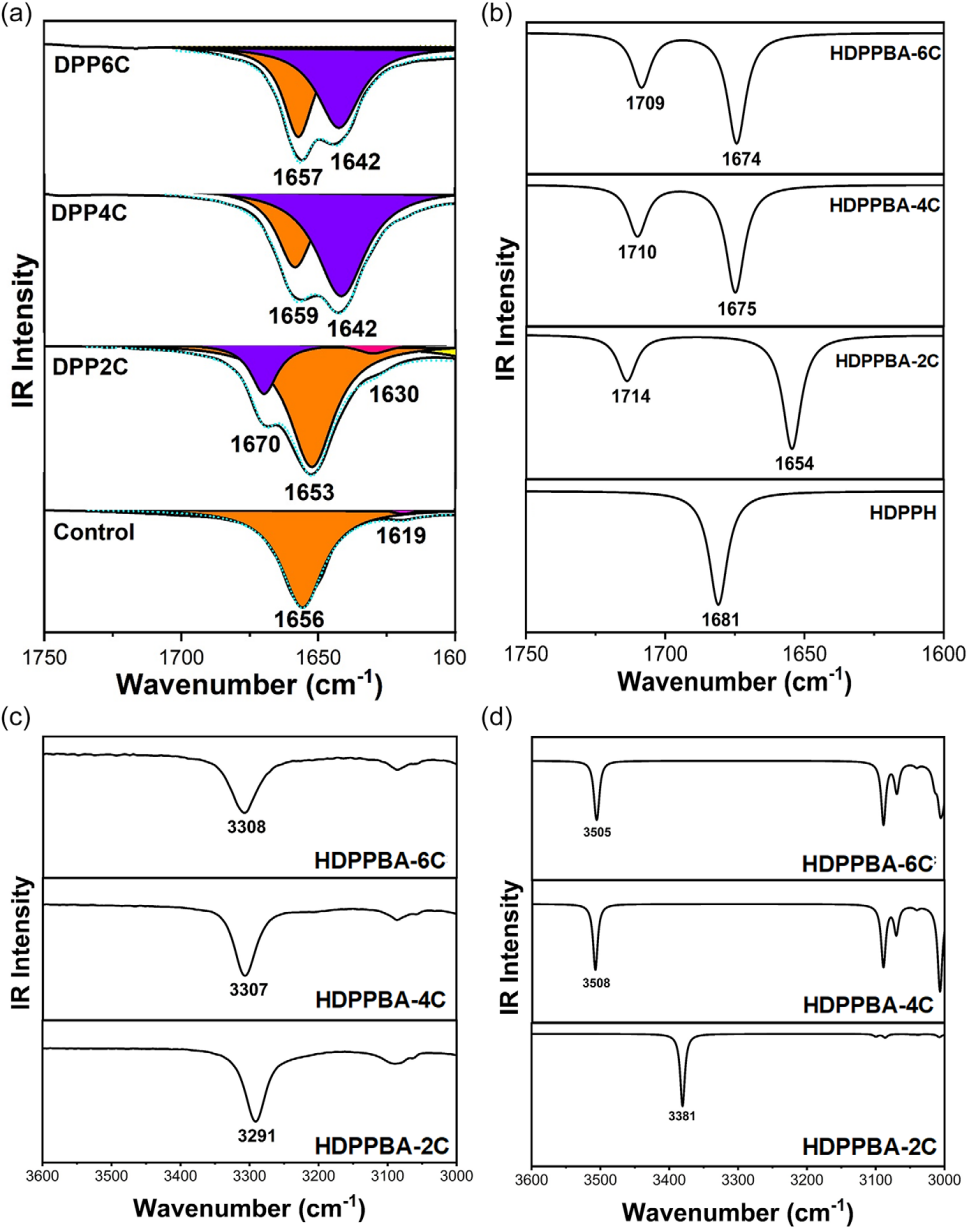
IR spectra of **HDPPBA‐2C**, **HDPPBA‐4C**, **HDPPBA‐6C,** and **HDPPH** in the carbonyl region: (a) experimental and (b) B3LYP/6‐31G** IR theoretical spectra. IR spectra in the amide region of **HDPPBA‐2C**, **HDPPBA‐4C,** and **HDPPBA‐6C**: (c) experimental and (d) B3LYP/6‐31G** IR theoretical spectra.

Vibrational spectroscopy was employed to experimentally identify hydrogen‐bond formation and assess its impact on molecular conjugation in the solid state. Attenuated total reflectance infrared (ATR‐IR) spectra of the stretching vibration of the C=O group (ν(C=O)), reveal two distinct C=O stretching contributions for the amide‐functionalized derivatives, corresponding to the carbonyl group in the DPP core and the pendant amide groups (Figure 4a, see the eigenvectors in Figure S7). **HDPPH** displays a unique IR band at 1656 cm^−1^ (Figure 4a), helping us to identify the ν(C=O) of the DPP core in the **HDPPBA** derivatives. Therefore, the DPP ν (C=O) IR band is recorded at 1653, 1659 and 1657 cm^−1^ for **HDPPBA‐2C**, **HDPPBA‐4C** and **HDPPBA‐6C**, respectively (Figure 4a). While the DPP carbonyl stretching frequency remains nearly invariant across the series, with a subtle shift towards higher wavenumbers found in **HDPPBA‐2C**, the amide C=O vibration exhibits a pronounced shift for **HDPPBA‐4C** and **HDPPBA‐6C** (1642 cm^−1^) relative to **HDPPBA‐2C** (1670 cm^−1^). This shift towards lower wavenumbers is indicative of strong hydrogen‐bond engagement of the amide carbonyls in the longer‐spacer derivatives, consistent with intermolecular hydrogen‐bonding. In contrast, the higher‐frequency amide C=O stretching observed for **HDPPBA‐2C** suggests the absence of strong intermolecular interactions involving the amide group. Theoretical DFT calculations (Figures 4b and S8) predict this IR band at around 1710 cm^−1^, which is consistent with the position found for **HDPPBA‐2C**. Thus, ATR‐IR data points out to the formation of intermolecular hydrogen‐bonding interactions in **HDPPBA‐4C** and **HDPPBA‐6C**, due to the weakening of the C=O bond of the amide group.

Analysis of the N–H stretching region further supports this assignment (Figure 4c,d). Experimental spectra show hydrogen‐bonded N–H vibrations for all three compounds (recorded around 3300 cm^−1^). However, DFT calculations (Figure 4d) predict significant N–H redshifting only for **HDPPBA‐2C** (3381 cm^−1^), consistent with intramolecular hydrogen‐bonding that can be captured at the single‐molecule level (Figure 4d), while for **HDPPBA‐4C** and **HDPPBA‐6C,** IR vibrations are predicted at much higher wavenumbers (∼3500 cm^−1^), indicating free amides (Figure 4d). These results point out that three compounds are involved in hydrogen‐bonding interactions but of different nature. Thus, **HDPPBA‐4C** and **HDPPBA‐6C** form intermolecular hydrogen‐bonding interactions, while the interactions in **HDPPBA‐2C** are intramolecular, occurring between the N–H group of the amide unit and the C=O group of the DPP unit, since theoretical calculations of a single molecule in vacuum are able to predict the interaction. The different nature of the hydrogen bonds in **HDPPBA‐4C** and **HDPPBA‐6C** versus **HDPPBA‐2C** also explains the shift toward higher wavenumbers of the ν (C=O) vibration of the amide in the latter, which is free of interaction, and the subtle shift of the ν (C=O) vibration of the DPP group, which is, in that case, involved in the intramolecular hydrogen‐bonding interactions (Figure 4a). The subtle shift toward lower wavenumbers is consistent with the formation of the hydrogen bond at the expense of breaking the intramolecular interaction between the thiophene hydrogen and the carbonyl oxygen (as shown in **HDPPBA‐6C** in Figure 1b) and accounts for the increased strength of the hydrogen bond versus the aforementioned intramolecular interaction.

The study of the hydrogen bond type can also be approached by the analysis of the relative intensities between the ν(C=O) of the amide and the DPP groups [27, 28]. IR intensity is primarily determined by dipolar moment changes during vibration; thus, hydrogen bonds formation should increase the IR ν(C=O) band intensity, as the interaction increases polarization of the C=O bond. As can be seen in Figure 4, for **HDPPBA‐2C**, the DPP ν(C=O) vibration is more intense than the amide one, thus pointing out to the formation of an intramolecular hydrogen bond, as discussed above. On the contrary, for **HDPPBA‐4C** and **HDPPBA‐6C**, the amide ν(C=O) vibration gains intensity with respect to the DPP one, being more pronounced in **HDPPBA‐4C**. These results point out the formation of intermolecular interactions for both systems, but these interactions are stronger in **HDPPBA‐4C** than in **HDPPBA‐6C.**

**FIGURE 5:**
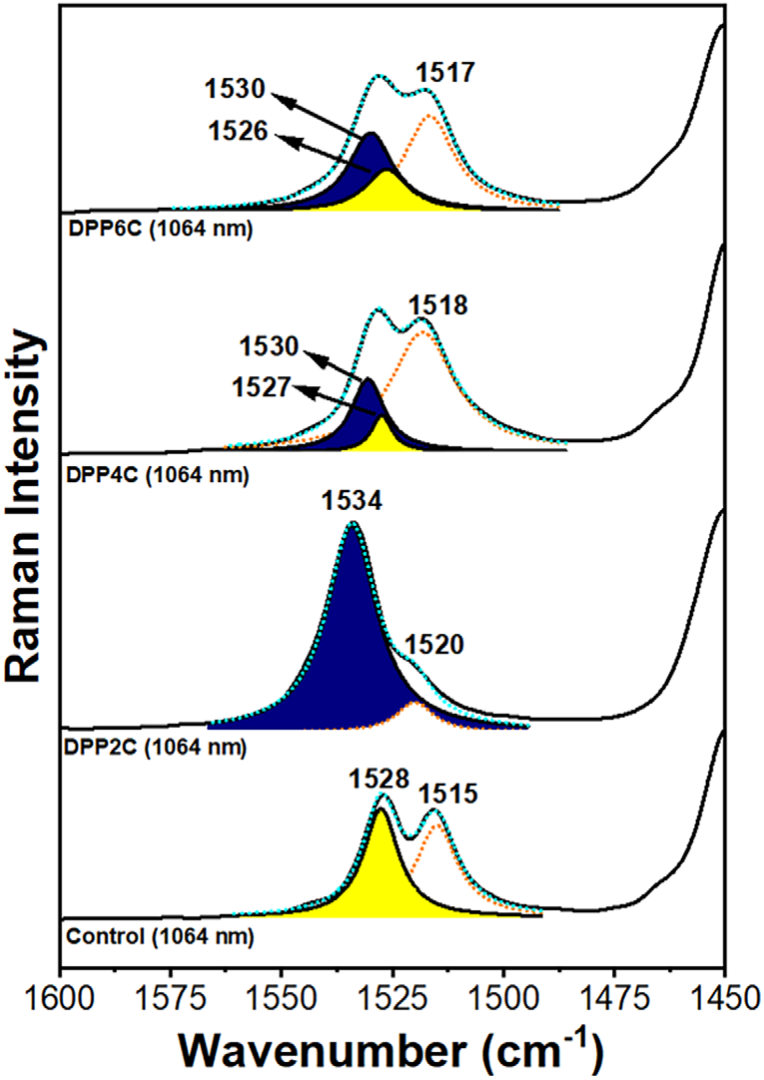
Raman spectra (1064 nm) of **HDDPH**, **HDPPBA‐2C**, **HDPPBA‐4C,** and **HDPPBA‐6C** as bulk samples.

Complementary insight into conjugation is provided by Raman spectroscopy, which probes the collective ν (C=C/C—C) vibration along the thiophene–DPP–thiophene backbone (Figure 5, see eigenvectors in Figure S9, and theoretical spectra in Figure S10). This Raman vibration is recorded at 1528 cm^−1^ for **HDPPH** (Figure 5), while it is slightly shifted toward higher wavenumbers (1534 cm^−1^) for **HDPPBA‐2C** (Figure 5), which is indicative of the loss of planarity due to the formation of intramolecular hydrogen‐bond interactions, as seen above. On the contrary, the position of this Raman band in **HDPPBA‐6C**, **HDPPBA‐4C**, where intermolecular hydrogen bonds are present, is comparable to that of the control molecule (1528 cm^−1^) but with a clear widening (Figure 5). Thus, two different contributions at 1526–1527 and 1530 cm^−1^ are found, indicative of the coexistence of various conjugation paths. This has been previously analyzed by our group [15] and is ascribed to some participation of the carbonyl group of the DPP unit in the supramolecular hydrogen‐bond interactions. In our previous work, this effect was attributed exclusively to an increased extension of π‐conjugation. However, given that hydrogen‐bond formation at the DPP carbonyl disrupts the planarity of the molecular backbone, both factors must be taken into account. Overall, vibrational spectroscopy corroborates the DFT results and demonstrates that hydrogen‐bond type governs both backbone planarity and effective conjugation in the solid state.

### 2.4 Electrochemical and Spectroelectrochemical Studies

**FIGURE 6:**
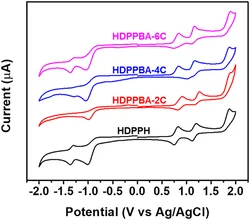
Cyclic voltammograms of **HDPPH**, **HDPPBA‐2C**, **HDPPBA‐4C,** and **HDPPBA‐6C**, (1 mM) full sweep of at 100 mV/s scan rate (referenced against potential of Fc/Fc^+^) in CH_2_Cl_2_ with tetrabutylammonium hexafluorophosphate 0.1 M as supporting electrolyte.

Cyclic voltammetry measurements in solution (1 mM in CH_2_Cl_2_) reveal similar redox behavior for all three derivatives, with two one‐electron oxidation processes and two reduction process observed within comparable potential windows (Figure 6). The HOMO and LUMO energy levels were calculated from the oxidation and reduction onset potentials, respectively and using ferrocene as a reference (Table S2).

The HOMO levels of **HDPPH**, **HDPPBA‐2C**, **HDPPBA‐4C,** and **HDPPBA‐6C** were calculated to be −5.1, −5.14, −5.1, and −5.09 eV, respectively. We observe modest shifts in oxidation potentials, except for **HDPPBA‐2C** which displays slightly higher values, being this consistent with its reduced backbone planarity and stabilized frontier orbitals predicted by DFT. On the other hand, the obtained LUMO values were −3.37, −3.39, −3.34, and −3.37 eV (see Supporting Informaton for details on the HOMO and LUMO calculations). The four derivatives have similar bandgaps, being 1.73, 1.75, 1.76, and 1.72 eV for **HDPPH**, **HDPPBA‐2C**, **HDPPBA‐4C,** and **HDPPBA‐6C**, respectively. Importantly, the overall similarity of the solution‐phase electrochemistry indicates that the intrinsic redox properties of the isolated molecules are largely unaffected by the position of the amide groups under these conditions, although this observation is consistent with the DFT‐predicted destabilization of the HOMO energy level in **HDPPBA‐2C** due to the formation of an intramolecular hydrogen bond interaction.

**FIGURE 7:**
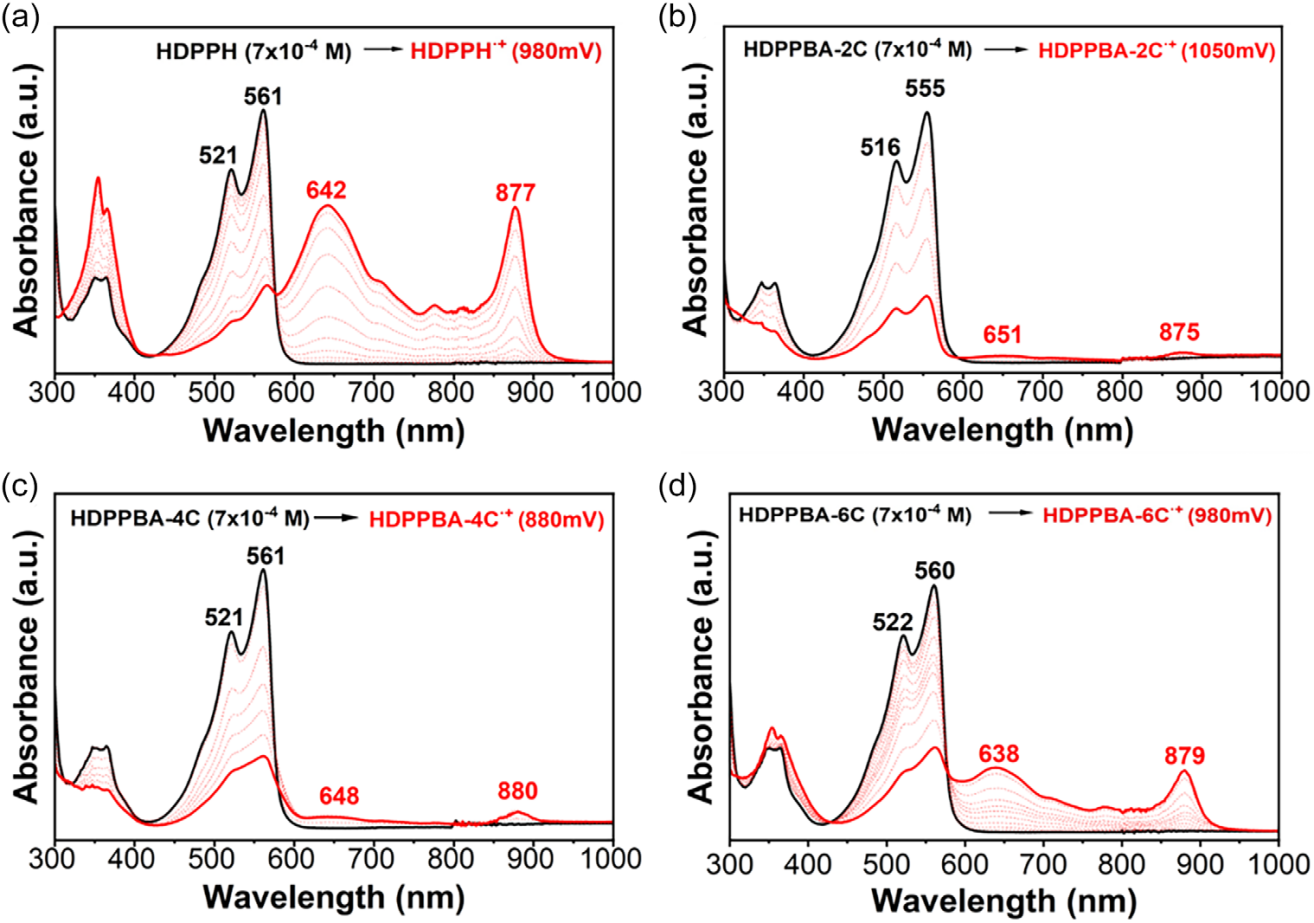
UV–vis–NIR absorption spectra of (a) **HDPPH**, (b) **HDPPBA‐2C**, (c) **HDPPBA‐4C,** and (d) **HDPPBA‐6C** electrochemically oxidized by progressive increase of the oxidation potentials.

Furthermore, in order to evaluate the ability of the studied semiconductors to stabilize charge carriers and to probe if the differences in hydrogen‐bonding type plays a role in the formation of charged species, we performed in situ spectroelectrochemical studies in dilute dichloromethane solution (10^−4^ M) at room temperature using 0.1 M Bu_4_NPF_6_ as supporting electrolyte and an optically transparent thin‐layer electrochemical (OTTLE) cell, as well as TD‐DFT calculations to support the experimental results (Figures S11–S14). As shown in Figure 7, **HDPPBA‐2C**, **HDPPBA‐4C**, **HDPPBA‐6C,** as well as the control **HDPPH** behave similarly in solution and can stabilize one positive charge. The similar behavior is expected due to the absence or minimization of intermolecular interactions in dilute solutions. Nevertheless, the distortion of the conjugated skeleton of **HDPPBA‐2C** due to the formation of intramolecular hydrogen bonds results in slightly higher potentials needed to stabilize the charges, which is in good agreement with the electrochemical results (Figure 6).

### 2.5 Electrical Studies

**FIGURE 8:**
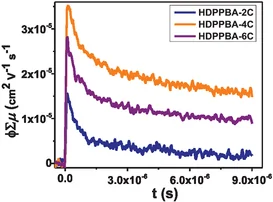
Kinetic traces of photoconductivity transients of **HDPPBA‐2C**, **HDPPBA‐4C,** and **HDPPBA‐6C** cast from CHCl_3_.

To directly probe charge‐carrier dynamics in the solid state, electrodeless photoconductivity measurements were performed using flash‐photolysis time‐resolved microwave conductivity (FP‐TRMC) [29, 30] on thin films of the three hydrogen‐bonded derivatives prepared by drop casting CHCl_3_ solutions of the DPP derivatives. This technique provides a measure of photoconductivity as *Φ*Σ*μ*, where *Φ* is the charge‐carrier generation quantum yield and Σ*μ* is the sum of charge‐carrier mobilities of electrons and holes (see Supporting Information for the measurement details). The thin films are irradiated with a pulsed laser generating charge carriers, being possible to follow their lifetimes before recombination. The kinetic traces of conductivity transients are shown in Figure 8, and the calculated values of photoconductivity (*φ*Σ*μ*), rate constants (*k*, calculated 3 µs after pulse excitation) and half lifetime (*t*_1/2_) are summarized in Table 1. All compounds exhibit measurable photoconductivity, ranging from 1.5 to 3.5 × 10^−5^ cm^2^ V^–1^ s^–1^ (Table 1), with **HDPPBA‐2C** being the derivative with the lowest photoconductivity value. Interestingly, remarkable differences emerge in charge‐carrier lifetimes. **HDPPBA‐4C** and **HDPPBA‐6C** display significantly longer carrier lifetimes up to one order of magnitude higher than **HDPPBA‐2C**, which has charge‐carrier lifetime values in the same order as **HDPPH** which is unable to form hydrogen‐bonds [16]. This indicates that while charge generation efficiencies are similar, charge stabilization and transport are markedly less effective in **HDPPBA‐2C**.

**TABLE 1:** Photoconductivity (*φ*∑*μ*) values, rate constants (*k*, calculated 3 µs after pulse excitation), and half lifetime (*t*_1/2_) values for HDPPBA‐2C, HDPPBA‐4C, and HDPPBA‐6C.

	*φ*∑*μ*, cm^2^V^−1^s^−1^	*k*, s^−1^	*t*_1/2_, s
HDPPBA‐2C	1.5 × 10^–5^	1 × 10^5^	5 × 10^−6^
HDPPBA‐4C	3.5 × 10^–5^	4 × 10^4^	2 × 10^−5^
HDPPBA‐6C	2.8 × 10^–5^	4 × 10^4^	2 × 10^−5^

This trend mirrors the results found on hydrogen‐bond topologies. In **HDPPBA‐4C** and **HDPPBA‐6C**, the presence of hydrogen‐bond‐stabilized electroactive populations facilitates not only charge formation but also charge persistence, consistent with delocalized conjugation pathways and interconnected supramolecular networks. Moreover, the stronger hydrogen‐bonding character in **HDPPBA‐4C**, as evidenced by IR spectroscopy, is consistent with the observed increase in photoconductivity (*φ*Σ*μ*) values. In contrast, the uniform but intramolecularly hydrogen‐bonded environment of **HDPPBA‐2C** enables oxidation but fails to support long‐lived, mobile charge carriers.

**FIGURE 9:**
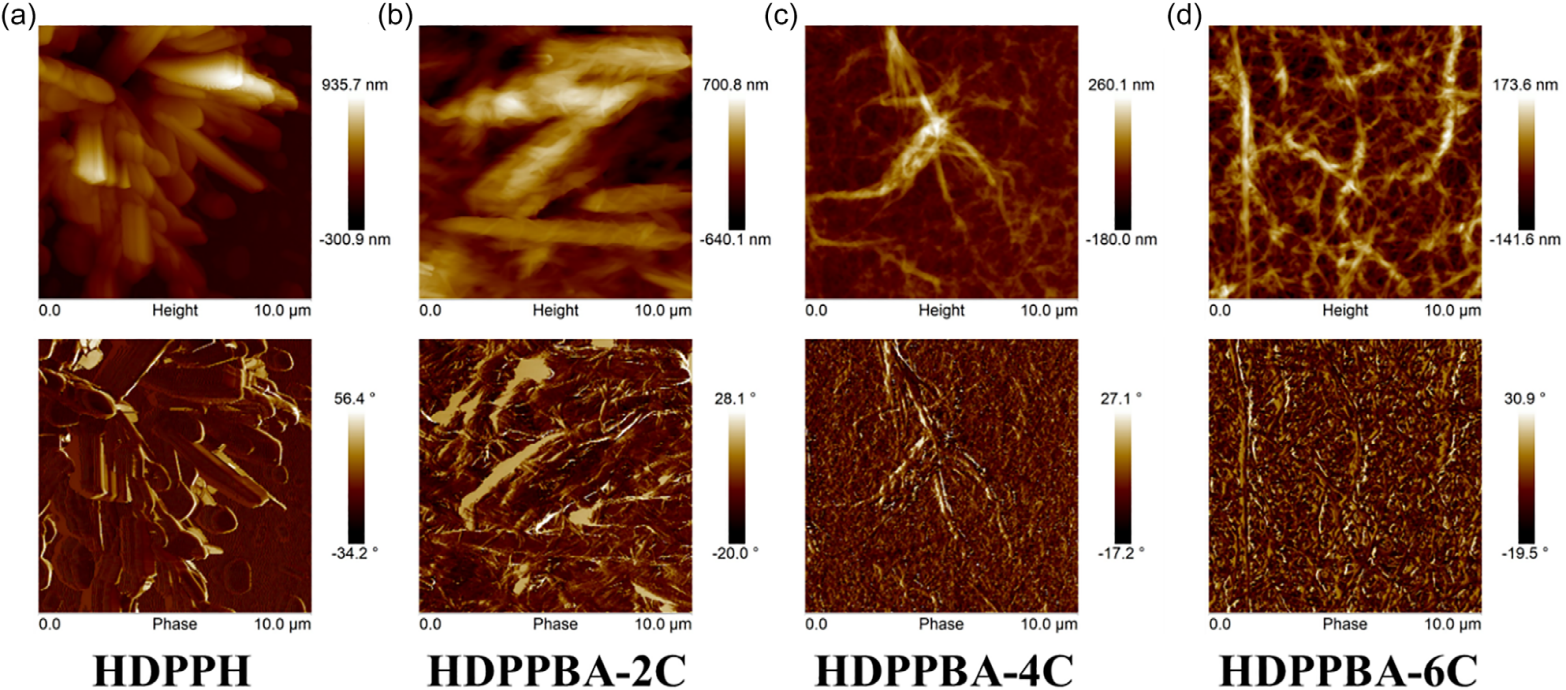
AFM of semiconducting thin films, (a) **HDDPH**, (b) **HDPPBA‐2C**, (c) **HDPPBA‐4C**, and (d) **HDPPBA‐6C**.

AFM, along with X‐ray diffraction (XRD), provides a structural basis for these observations (Figures 9 and S13). AFM images show that **HDPPBA‐4C** and **HDPPBA‐6C** form dense, interconnected fibrillar networks, providing continuous pathways for charge transport. This highly interconnected morphology correlates with the absence of long‐range ordering in the low‐angle XRD profiles of the **HDPPBA‐4C** and **HDPPBA‐6C** derivatives. The lack of sharp diffraction peaks in this region suggests a more amorphous molecular arrangement in the solid state, which potentially results in the growth of interconnected fibrillar networks. In contrast, the AFM images of **HDPPBA‐2C** show that it self‐assembles into shorter, poorly connected domains, which aligns with the diffraction peak observed at low angle. This reflection indicates the preservation of a rigid stacking that favors localized molecular aggregation over extended fibrillar network formation. Consequently, these shorter and poorly connected domains act as barriers to long‐range charge migration and promote rapid recombination. Thus, FP‐TRMC, AFM, and PXRD together demonstrate that hydrogen‐bond type governs not only electronic energetics but also mesoscale organization, both of which are essential for efficient charge transport.

Even though the FP‐TRMC measurements give information on charge transport at the nanoscale, these results are very valuable to predict the performance of the different derivatives in lab‐scale devices.

Solution‐processed OFET transistors with a top‐contact/bottom‐gate structure were also fabricated as described below. From the saturation region in the device transfer curves, the field effect mobility (*µ*), threshold voltage (*V*_T_), and on/off current ratio (*I*_ON_/*I*_OFF_) were determined. The electrical parameters of the best‐performing devices are summarized in Table 2.

**TABLE 2:** OFET electrical data for best‐performing deposited films of the indicated semiconductors measured in vacuum.

Compound	Subst.^a^ treatment	*μ*_h_, cm^2 ^V^−1 ^S^−1^	*V*_T_, V	*I*_ON_/*I*_OFF_
HDPPH	OST (120 °C)	NA	NA	NA
HDPPBA‐2C	OST (120 °C)	4 × 10^−5^ ± 7 × 10^−6^	−10	4 × 10^+1^
HDPPBA‐4C	OST (120 °C)	5 × 10^−3^ ± 7 × 10^−4^	−15	3 × 10^+3^
HDPPBA‐6C	OST (120 °C)	2 × 10^−2^ ± 2 × 10^−3^	−12	2 × 10^+4^

^a^ Substrates treated with OTS and thermal annealed at 120 °C.

**FIGURE 10:**
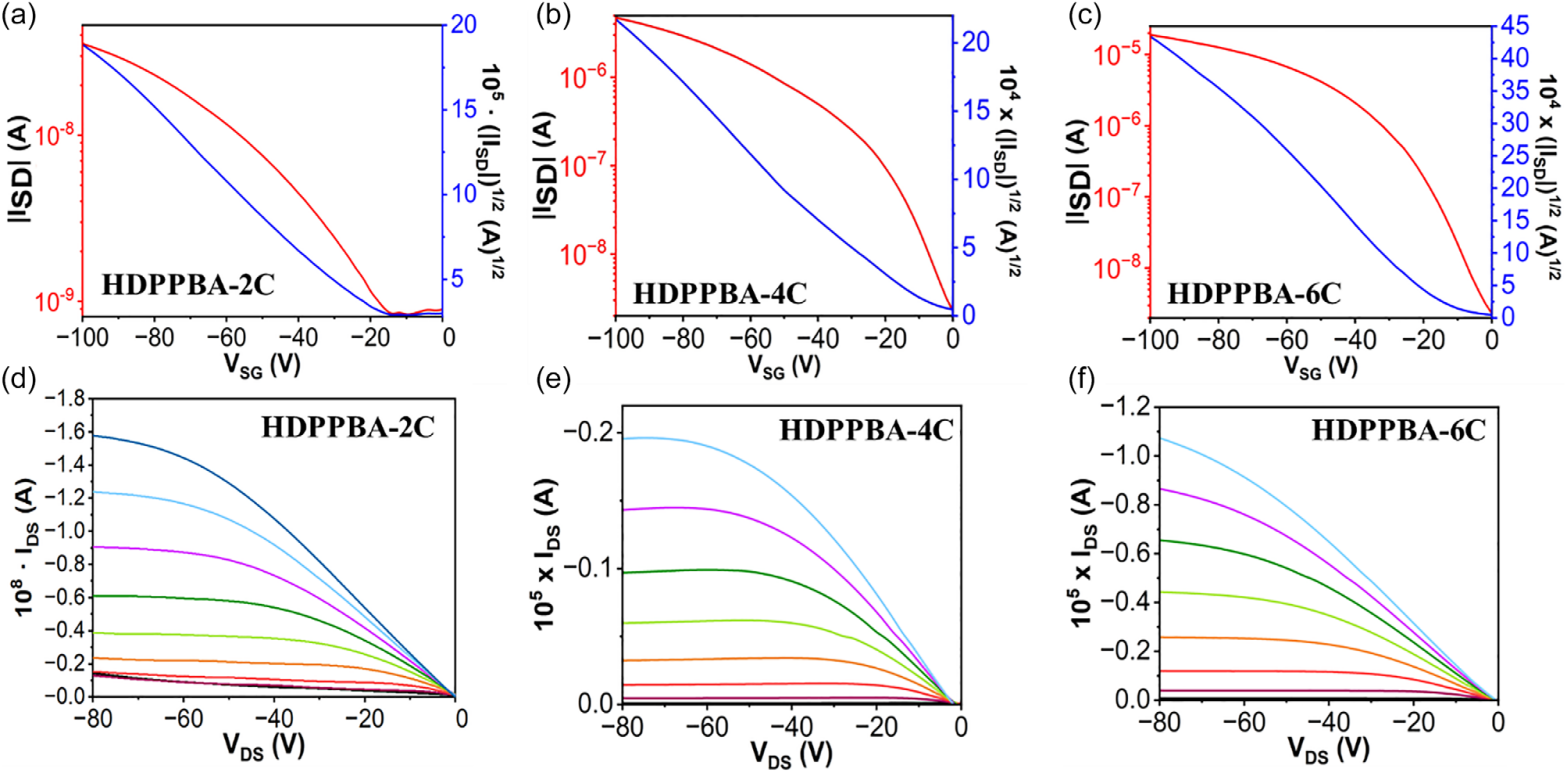
Transfer (a–c) and output (d–f) of **HDPPBA‐2C**, **HDPPBA‐4C,** and **HDPPBA‐6C**. The transfer characteristics were measured at a constant source–drain voltage of −80 V. The gate voltage in the output plots varies from 0 to −80 V in steps of 10 V.

Thin films of **HDPPH**, **HDPPBA‐2C**, **HDPPBA‐4C,** and **HDPPBA‐6C** were deposited by drop casting from chloroform solutions (3 mg/mL) onto Si/SiO_2_ substrates. After deposition, thermal annealing was carried out at 120 °C for 3 h to promote molecular organization and improve the film quality. Notably, while **HDPPH** showed no activity in OFETs, p‐type field‐effect mobilities of 4 × 10^−6^, 5 × 10^−3^, and 2 × 10^−2^ cm^2^ V^−1^ s^−1^ were obtained for **HDPPBA‐2C**, **HDPPBA‐4C,** and **HDPPBA‐6C,** respectively (see output and transfer plots in Figure 10 and the full characterization, with at least five devices measured for each material, in Table S3 and Figure S15 for PXRD of the active layers). Thus, the field effect mobilities increase with the elongation of the alkyl spacer between the amide groups and the DPP core. This is probably due to the presence of intermolecular hydrogen bonds in **HDPPBA‐4C** and **HDPPBA‐6C**, which modifies thin film morphology, as shown in AFM images, thus favoring charge transport. Note also that the participation of the carbonyl group of the DPP unit in the hydrogen‐bonds formation, as previously demonstrated by our group, also influences π‐conjugation, facilitating charge injection and transport. Furthermore, the differences observed between the **HDPPBA‐4C** and **HDPPBA‐6C** semiconductors may be associated with the stronger hydrogen‐bonding interactions in the former, as evidenced by spectroscopy, which may hinder thin‐film optimization during the annealing process. Accordingly, Table S3 shows a smaller impact of thermal annealing on **HDPPBA‐4C** compared to **HDPPBA‐6C**.

In contrast, in **HDPPBA‐2C** the proximity of the amide group to the conjugated backbone favors intramolecular hydrogen bonds, which has two effects: (i) inhibit the formation of hydrogen‐bonded supramolecular aggregates and (ii) has a clear impact on the molecular structure since the molecular skeleton losses planarity, decreasing effective conjugation, and thus, slightly diminishing the ability to inject and stabilize charge carriers (as seen in Section 2.4). These results corroborate that modifying the position of amide groups on side chains is a highly effective strategy for promoting π‐conjugation, stabilizing charged species and generating supramolecular aggregates.

## 3 Conclusions

This study demonstrates that hydrogen‐bond type and strength, rather than hydrogen‐bonding per se, governs conjugation pathways, charge stabilization, and charge transport in DPP‐based small‐molecule semiconductors. Precise control over the spatial positioning of amide functionalities enables selective promotion of intra‐ or intermolecular hydrogen‐bonding, with important consequences for molecular planarity, supramolecular organization, and electronic coupling in the solid state. Proximal amide groups induce intramolecular hydrogen‐bonding that disrupts backbone planarity, restricts effective π‐conjugation, and limits the formation of electronically connected domains, leading to slightly higher oxidation onsets, short‐lived charge carriers, and poor device performance. In contrast, distal amide placement favors intermolecular hydrogen‐bonding involving the DPP carbonyl groups, stabilizing planar conformations, extending conjugation pathways across neighboring molecules, and generating heterogeneous but electronically delocalized environments capable of stabilizing long‐lived charges. These effects are reflected in slightly lowered oxidation energetics, enhanced photoconductivity lifetimes, and significantly improved field‐effect mobilities. Taken together, these findings establish hydrogen‐bond topology as a powerful and general design principle for engineering the electronic structure and charge transport properties of supramolecular organic semiconductors.

## Funding

This work was supported by Ministerio de Ciencia e Innovación (Grants PID2022‐139548GB‐I00 and PID2025‐172387OB‐I00), Ministerio de Ciencia, Innovación y Universidades (Grant ATR2024‐154740), Agence Nationale de la Recherche (Grants ANR JCJC TOTALBOND 2020 and CSC‐IGSANR‐17‐EURE‐0016), Junta de Andalucía (Grant FQM‐159), and University of Málaga (Grant PPRO‐FQM159‐G‐2023).

## Conflicts of Interest

The authors declare no conflicts of interest.

## Supporting information

Supplementary Material

## Data Availability

The data that support the findings of this study are available in the Supporting Information of this article.
