# Supplementary material for: Tuning Conjugation and Charge Transport in Supramolecular Semiconductors via Hydrogen‐Bonding Position
Source: Small Sci. 2026 Aug 17;6(8):e70383. doi: 10.1002/smsc.70383 (PMC13489308; doi:10.1002/smsc.70383)
Supplement: Supplementary file 1 [file SMSC-6-e70383-s001.pdf]

Tuning Conjugation and Charge Transport in Supramolecular Semiconductors via Hydrogen-Bonding Position

[a]Raúl Gónzalez-Núñez, [b]Gabriel Martinez, [a]Kyeong-Im Hong, [c]Wakana Matsuda, [c]Shu Seki, [d]Pablo Lupiáñez-Garrido, [a,b*]Amparo Ruiz-Carretero, [d,e*]Rocío Ponce Ortiz

[a] Instituto de Ciencia de Materiales de Madrid, Consejo Superior de Investigaciones Científicas CSIC, Sor Juana Inés de la Cruz 3, 28049, Madrid, Spain

[b]University of Strasbourg, Institute Charles Sadron, CNRS, UPR22, 23 Rue du Loess, 67034, Strasbourg Cedex 2, France

[c]Department of Molecular Engineering, Graduate School of Engineering, Kyoto University, Nishikyo-ku, Kyoto 615-8510, Japan

[d]Department of Physical Chemistry, University of Málaga, Campus de Teatinos s/n, Málaga 29071, Spain.

[e]Instituto Universitario de Materiales y Nanotecnología, IMANA, University of Málaga, Campus de Teatinos, 29071, Málaga, Spain

*Corresponding author

E-mail: amparo.ruiz@csic.es; rocioponce@uma.es

Table of contents

Experimental section……………………………………………………………………………..2

Synthetic protocols and characterization………………………………………………………….5

DFT and TD-DFT calculations………………………………………………………..…….15

Vibrational study……………………………………………………………………………….…….22

Electrochemical data……………………………………………………………………….…...26

Spectroelectrochemistry ………………………………………………………………….....26

Field effects transistors characterization……………………………………………..……..28

References……...……………………………………………………………………………….….31

Experimental section

All reagents and solvents were obtained from commercial suppliers and purified or dried according to standard procedures. Column chromatography was performed on silica gel (VWR Silica 60, particle size 0.040–0.063 mm). Solvents for spectroscopic studies were of spectroscopic grade and used as received. The high-resolution mass was obtained in a MICROTOF II focus (Bruker) with an ESI (electro spray ionization) source. The software used for ion detection is Package Compass Data Analysis (Bruker Daltonics, Bremen, Germany).

^{1}H and ^{13}C spectra were recorded in $CDCl_3$ and Tol-d8 on a Bruker Avance 400 MHz spectrometer and/or Bruker Avance III HD 500 MHz spectrometer. UV–vis measurements were performed in a conventional quartz cell (light pass 1 mm) or on thin film cast on glass substrates on a Cary 5000 UV-

Vis-NIR spectrophotometer. The variable temperature (VT) measurements were performed in a Bruker-300 Ultra Shield spectrometer (300 MHz for ^1H and 75 MHz for ^{13}C).

FTIR spectra were recorded with a Vertex 70 from Bruker Optics, equipped with MCT detector and a black-body source. The spectra of the solids were measured by ATR on diamond. The solutions were studied in cells from Specac Pike with KBr NaCl windows. The spectra were compensated from CO_2 and moisture with OPUS from Bruker. The solvent intensities were measured separately and subtracted.

Cyclic voltammetry

Cyclic voltammetry measurements were carried out under an atmosphere of argon using a three-electrode arrangement in a single compartment cell. A platinum disc working electrode, a platinum wire counter electrode and a non-aqueous Ag/Ag^+ reference electrode were used in the cell using an Ossila potentiostat. Redox potentials are quoted versus the ferrocenium-ferrocene couple, which was used as an internal reference, 100 mV/s scan rate. CH_2Cl_2 (dried and degassed) was utilized as solvent with tetrabutylammonium hexafluorophosphate ($TBAPF_6$, 0.1 M) employed as supporting electrolyte**.**

HOMO and LUMO levels were calculated using the equations:

$E_{HOMO} = -(E_{onset}(ox) + 4.8\ [eV])$ and $E_{LUMO} = -(E_{onset}(red) + 4.8\ [eV])$.

The band gap is calculated using the equation

$Eg = E_{HOMO} - E_{LUMO}\ [eV]$.

Flash-Photolysis Time-Resolved Microwave Conductivity

Charge carrier mobilities were evaluated by FP-TRMC at room temperature. The thin films of samples were fabricated by drop-cast method onto quartz substrates ($9 \times 40\ mm^2$, 1 mm thick). Charge carriers were injected into the materials via photo-ionization with a third harmonic generation ($\lambda = 355$ nm) of a Spectra Physics model INDI-HG Nd:YAG laser pulses at 10 Hz with a pulse duration of ca. 5 ns. The photon density of a 355 nm pulse was modulated from 9.1×10^{15} photons cm^{-2} $pulse^{-1}$. A GUNN diode oscillator model MMP-3015 (Nakadai S.S. Japan) was used as the microwave power source. The microwave frequency and power were set at ~9.1 GHz and 3 mW,

respectively, and guided into a microwave cavity. The Q-factor of the microwave cavity loaded with the sample was 2200, and the substrates with the compound films were set at the point of electric field maximum. The reflected power of the probing microwave, picked up by a GaAs crystal–diode with Schottky–barriers (rise time < 1 ns), was monitored by a Tektronics model TDS3054 digital oscilloscope. The observed change in the reflected microwave power (ΔP_r) was normalised with the steady reflection of the microwave from the cavity (P_r), and converted directly into the product of a photocarrier generation yield (ϕ) and the sum of photo-generated electron/hole mobilities ($\Sigma\mu$),

$$\phi\Sigma\mu = 1/(eI_0F_{light}) \times (1/A) \times (\Delta P_r/P_r), \quad (1)$$

where e, A, I_0, and F_{light} are elementary charge, sensitivity factor (S^{-1} cm), incident photon density of the excitation laser (photon cm^{-2}), and filling factor (cm^{-1}), respectively. The value of F_{light} was calculated based on the overlap of the area of photo-carrier injection (presumed to be proportional to the absorbance of excitation light by the sample film) with electric field strength distribution in the cavity derived from a calculation code of CST Microwave Studio from AET Inc.

Synthetic protocols and characterization

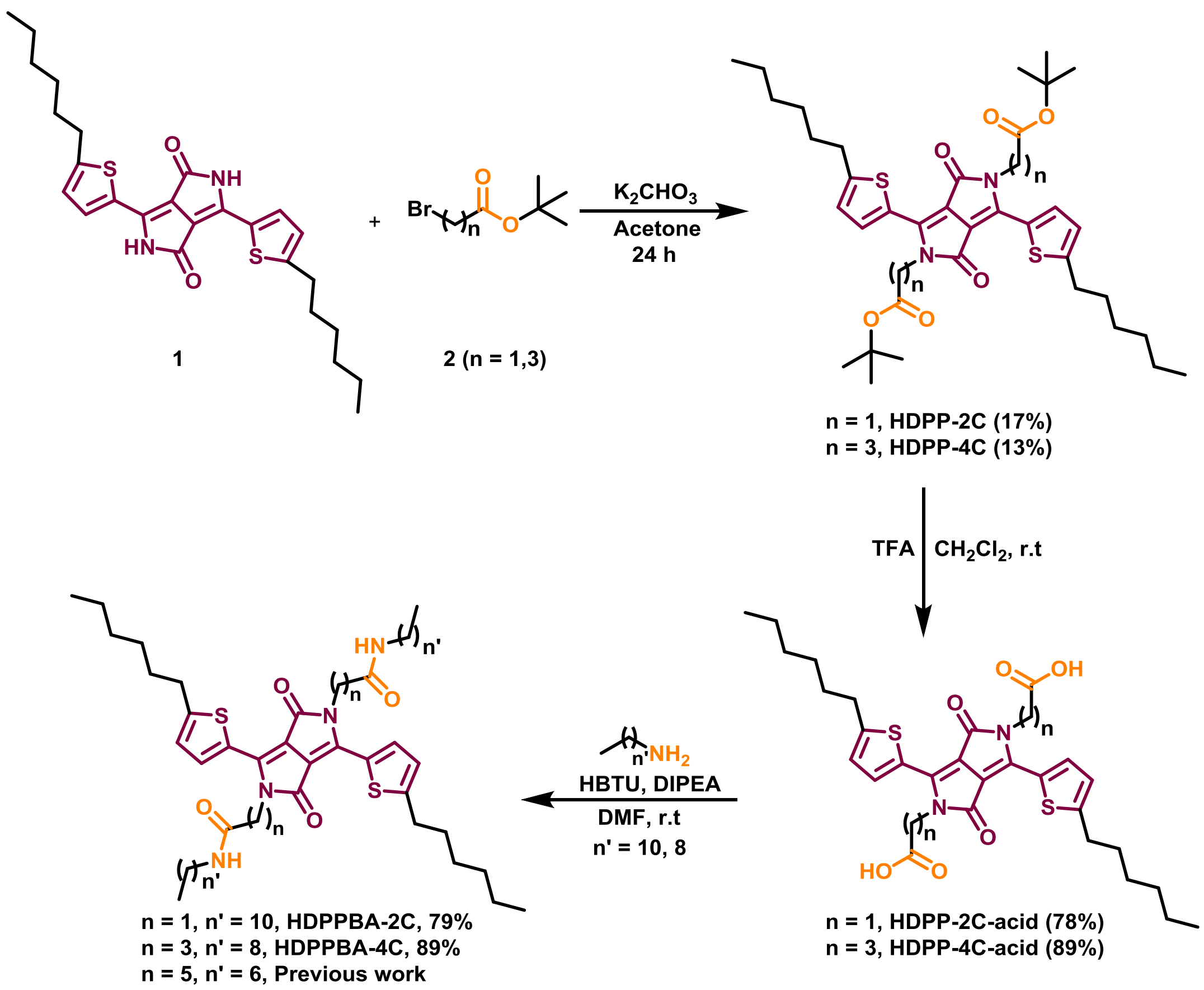

Scheme S1. Synthetic route towards **HDPPBA-2C** and **HDPPBA-4C**.

HDPP-2C **-** Di-tert-butyl 2,2'-(3,6-bis(5-hexylthiophen-2-yl)-1,4-dioxopyrrolo[3,4-c]pyrrole-2,5(1H,4H)-diyl)diacetate

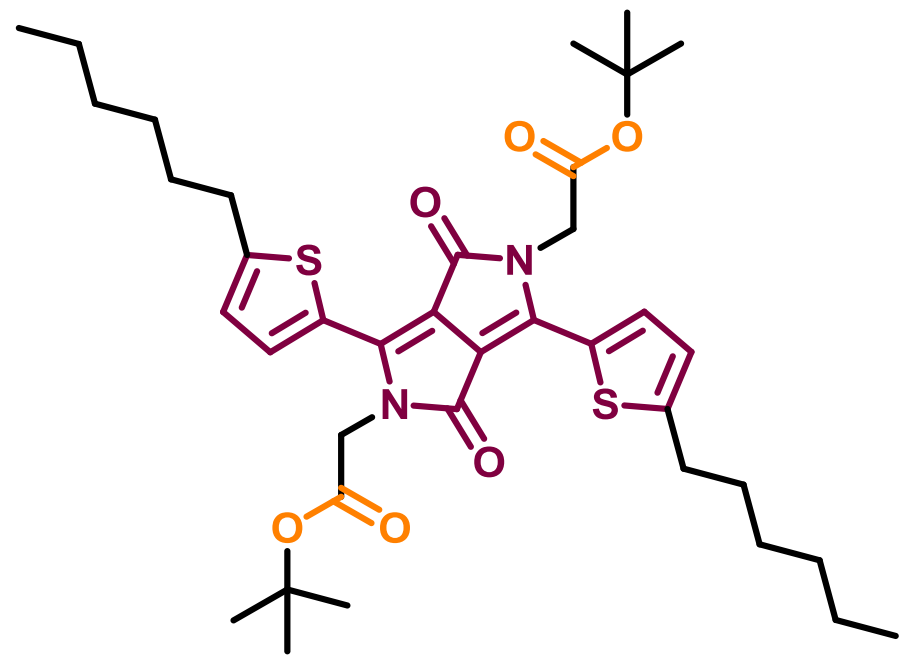

K_2CO_3 (1.3852 g, 10.0 mmol) and tert-butyl bromoacetate (1.0 mL, 6.8 mmol) were added to a suspension of **1** (1.1109 g, 2.4 mmol) in acetone (25 mL). The mixture was heated under reflux and stirred overnight under argon atmosphere. The reaction was allowed to cool down to room temperature and the solvent was evaporated under reduced pressure. The residue was dissolved in $CHCl_3$ (70 mL) and washed with water (4x 70 mL). The organic phase was dried over $MgSO_4$, filtered and evaporated to dryness under vacuum. The crude product was purified by column chromatography using toluene-methanol (1.0 % up to 2.0%) as eluent. The desired product was obtained as a dark violet solid (0.2736 g, 17%).

^{1}H NMR (400 MHz, $CDCl_3$) δ[ppm]: 8.59 (d, J = 3.9 Hz, 2H, ArH), 6.94 (d, J = 3.9 Hz, 2H , ArH), 4.77 (s, 4H, N-CH_2-), 2.87 (t, J = 7.6 Hz, 4H, Thiophene-CH_2-), 1.69 (p, J = 7.5 Hz, 4H, -CH_2-), 1.42 (s, 18H, OC-CH_3), 1.29 (m, 12H, -CH_2-), 0.88 (t, J = 6.8 Hz, 6H, -CH_3).

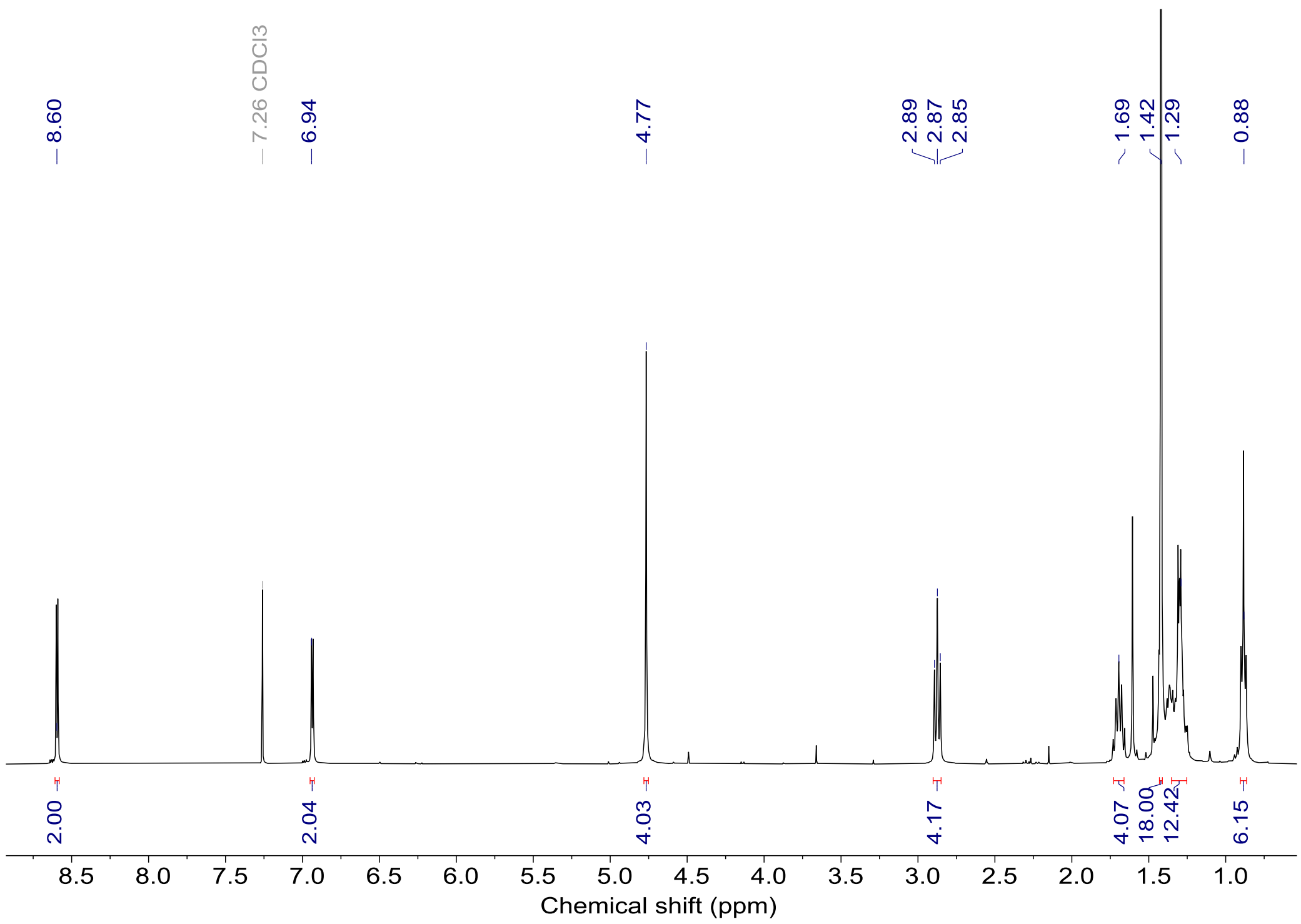

HDPP-2C-acid - 2,2'-(3,6-bis(5-hexylthiophen-2-yl)-1,4-dioxopyrrolo[3,4-c]pyrrole-2,5(1H,4H)-diyl)diacetic acid

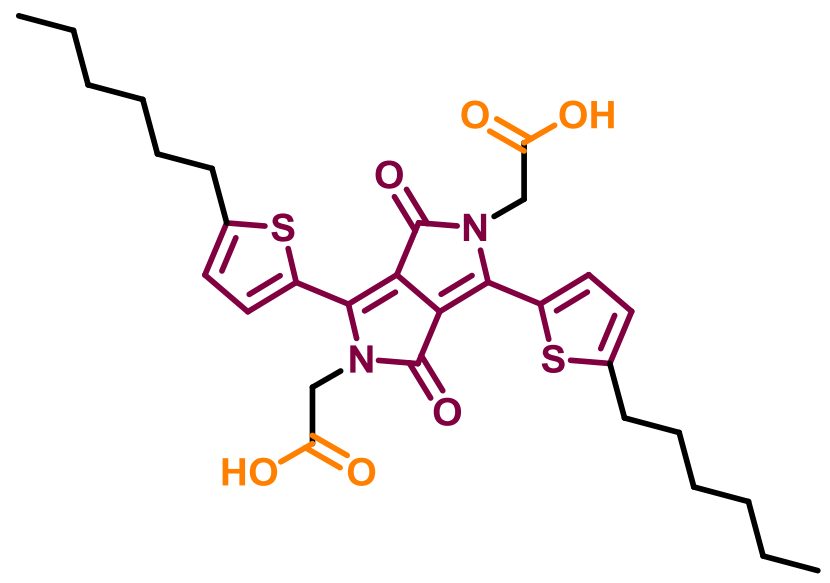

HDPP-2C (0.2736 g, 0.4 mmol) was dissolved in dichloromethane (8 mL) at room temperature. Then, trifluoroacetic acid was added (1.4 mL) and the mixture was stirred overnight. Next, ethyl ether (100 mL) was added to the reaction and it was stirred for 20 minutes. After filtrating the obtained solid was washed with ethyl ether several times. Following drying under reduced pressure the target compound was afforded as a dark violet solid (0.1781, 78%) in sufficient purity.

^{1}H NMR (400 MHz, DMSO) δ[ppm]: 13.42 (s, 2H, -O*H*), 8.53 (d, *J* = 3.9 Hz, 2H, Ar*H*), 7.15 (d, *J* = 3.9 Hz, 2H , Ar*H*), 4.73 (s, 4H , N-CH_2-), 2.89 (t, *J* = 7.6 Hz, 4H, Thiophene-CH_2-), 1.65 (p, *J* = 7.5 Hz, 4H, -CH_2-), 1.36 – 1.25 (m, 12H, -CH_2-), 0.86 (t, *J* = 2.5 Hz, 6H, -CH_3).

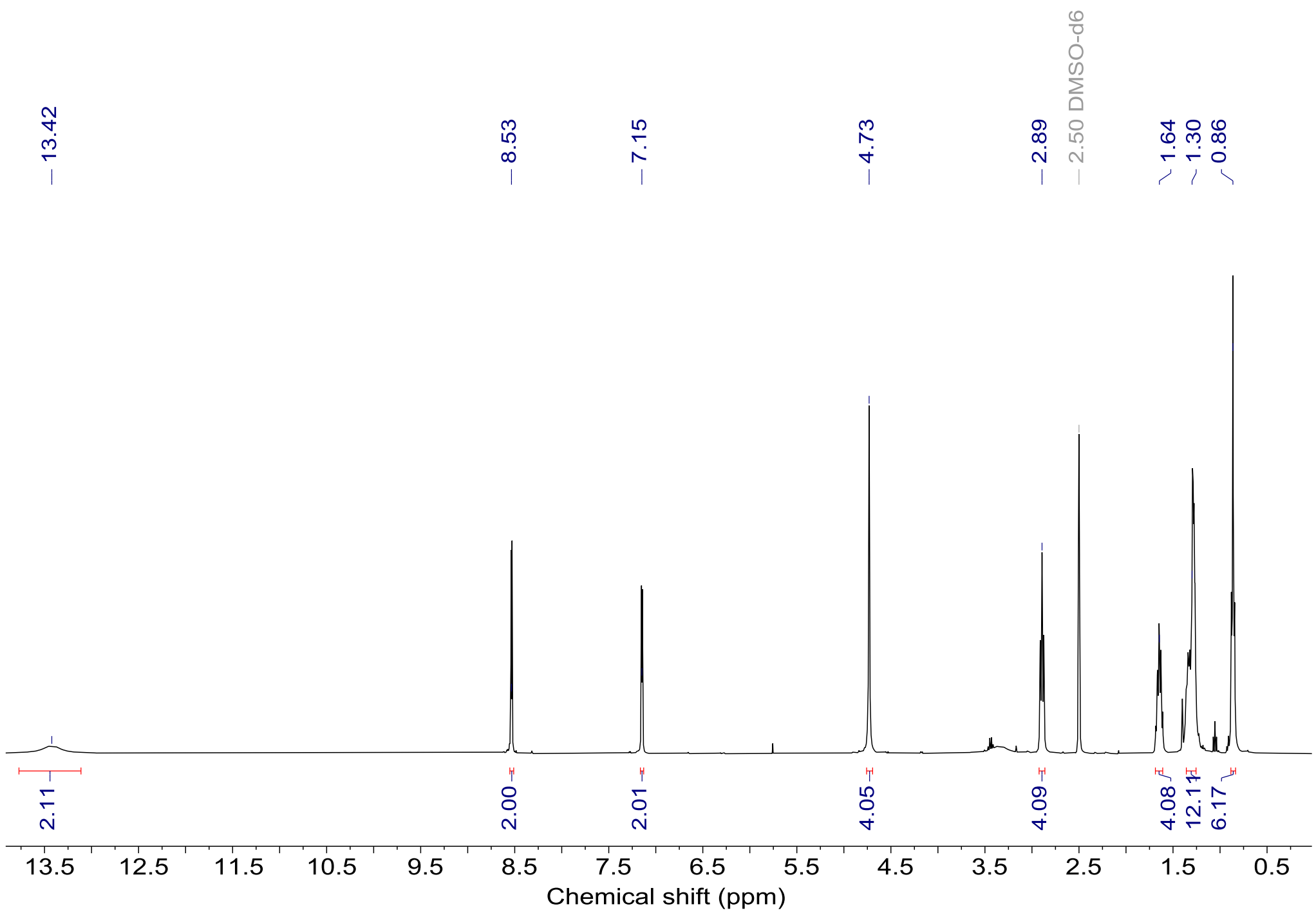

HDPPBA-2C 2,2'-(3,6-bis(5-hexylthiophen-2-yl)-1,4-dioxopyrrolo[3,4-c]pyrrole-2,5(1H,4H)-diyl)bis(N-decylacetamide)

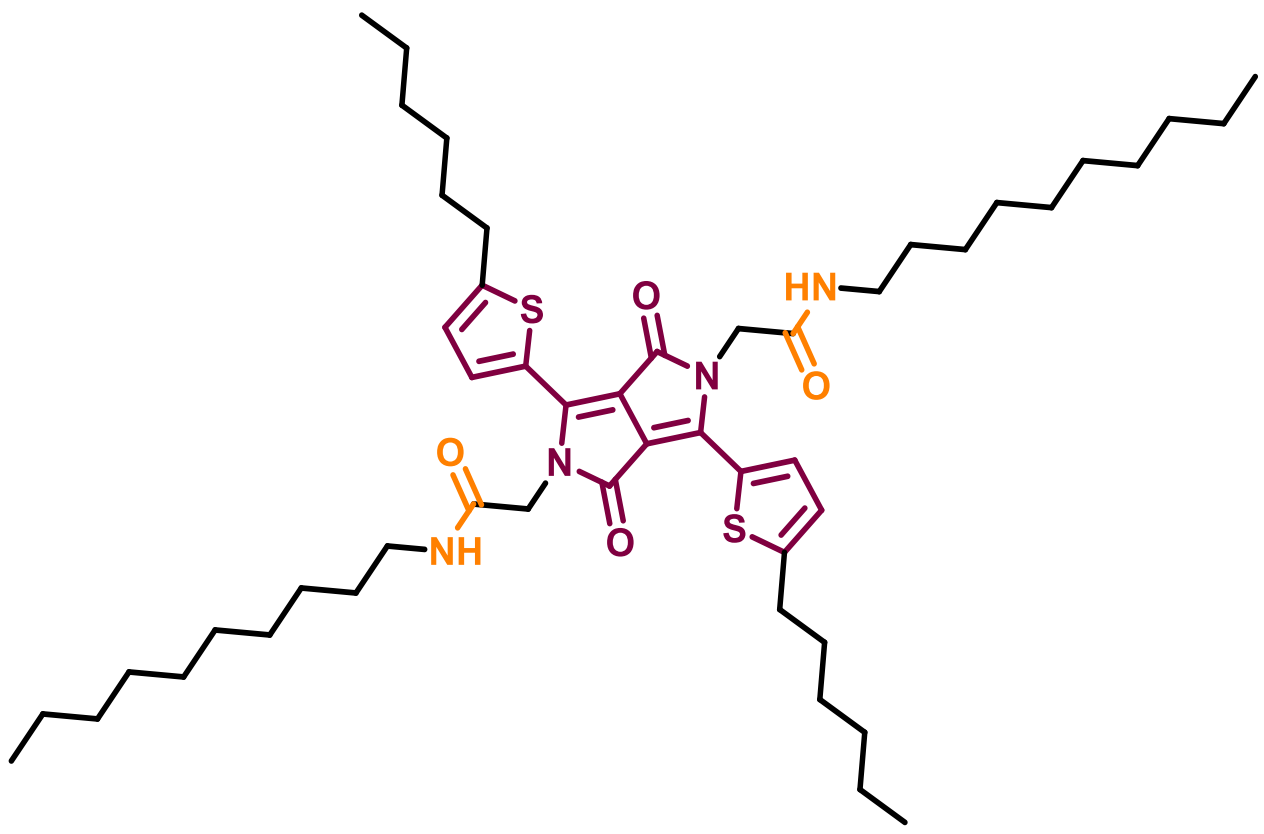

HDPP-2C-acid (0.1652 g, 0.28 mmol), HBTU (0.3832 g, 1.0 mmol) and decylamine (0.25 mL, 1.2 mmol) were dissolved in dry DMF (5 mL) under argon while stirring. After 5 min DIPEA (0.3 mL, 1.7 mmol) was added to the reaction mixture and it was stirred under argon atmosphere overnight at room temperature. After 17 h, the starting material was still visible by TLC therefore two more equivalents of decylamine, HBTU and DIPEA were added to the reaction and it was let react for 7 h more. The DMF was removed under vacuum at 70 °C. Then, the remaining solid was dissolved in the

minimum volume of hot $CHCl_3$ and poured in diethyl ether (200 mL). The resulting solid was washed with diethyl ether and then recrystallized again in a $CHCl_3$-methanol mixture to obtain the desired pure product as reddish violet solid (0.1920 g, 79%).

^{1}H NMR (400 MHz, $CDCl_3$) δ[ppm]: 8.49 (d, *J* = 3.9 Hz, 2H, Ar*H*), 6.96 (d, *J* = 4.0 Hz, 2H, Ar*H*), 6.14 (t, *J* = 6.0 Hz, 2H, N*H*), 4.68 (s, 4H, N-CH_2-), 3.25 (q, *J* = 6.8 Hz, 4H, -CONH-CH_2), 2.88 (t, *J* = 7.7 Hz, 4H, Thiophene-CH_2), 1.71 (p, *J* = 7.6 Hz, 4H, -CH_2-), 1.49 – 1.18 (m, 44H, -CH_2-), 0.95 – 0.83 (m, 12H, CH_2-CH_3).

^{13}C NMR (101 MHz, $CDCl_3$, 373 K) δ 167.5, 161.6, 154.8, 140, 135.4, 127.1, 126.8, 106.7, 46.46, 39.9, 32, 31.6, 31.6, 30.6, 29.7, 29.7, 29.6, 29.4, 29.4, 28.9, 26.9, 22.8, 22.7, 14.3, 14.2.

Theoretical $[M+K]^+$: 901.5137, HR-MS (ESI-TOF): 901.5068 $[M+K]^+$.

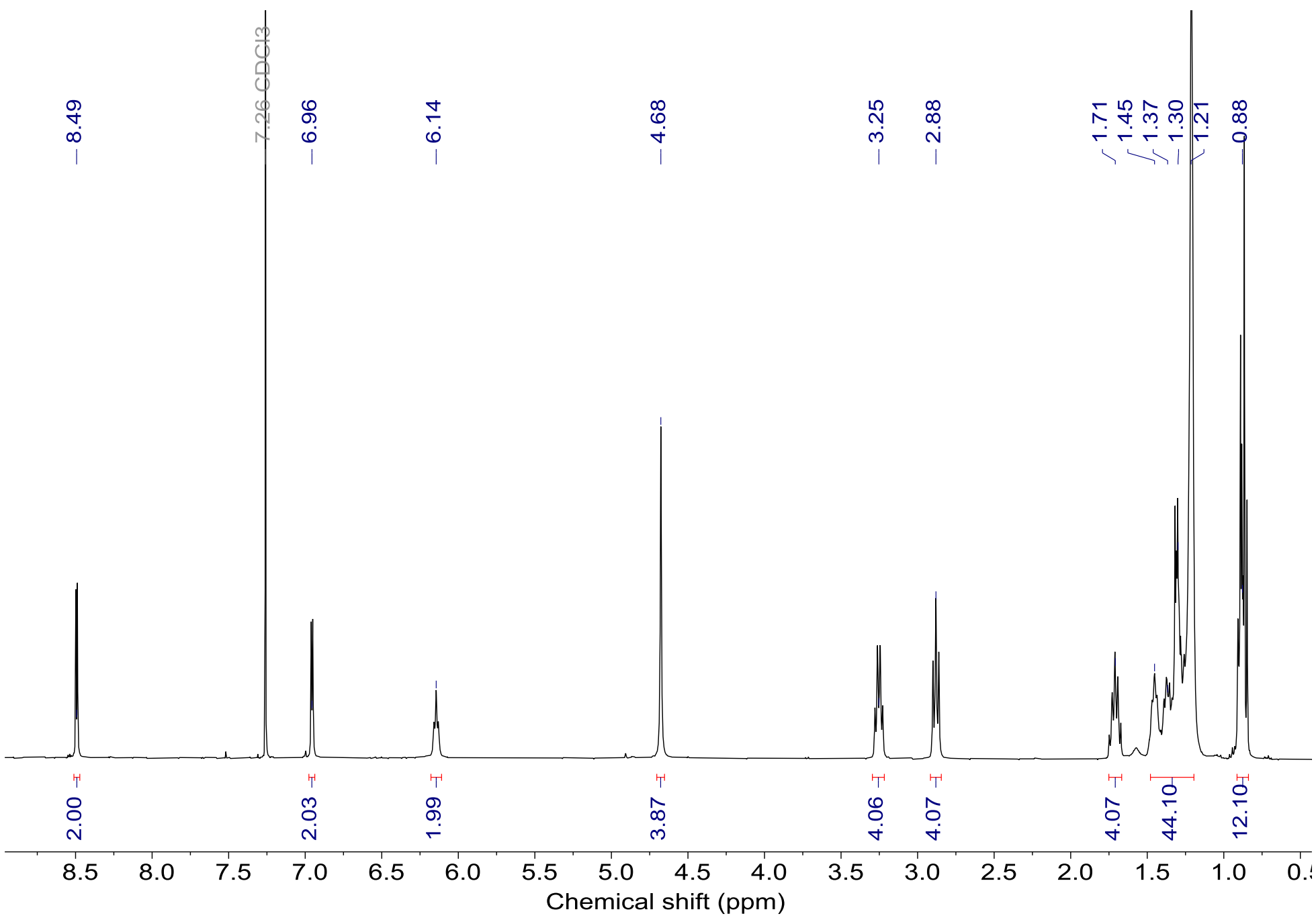

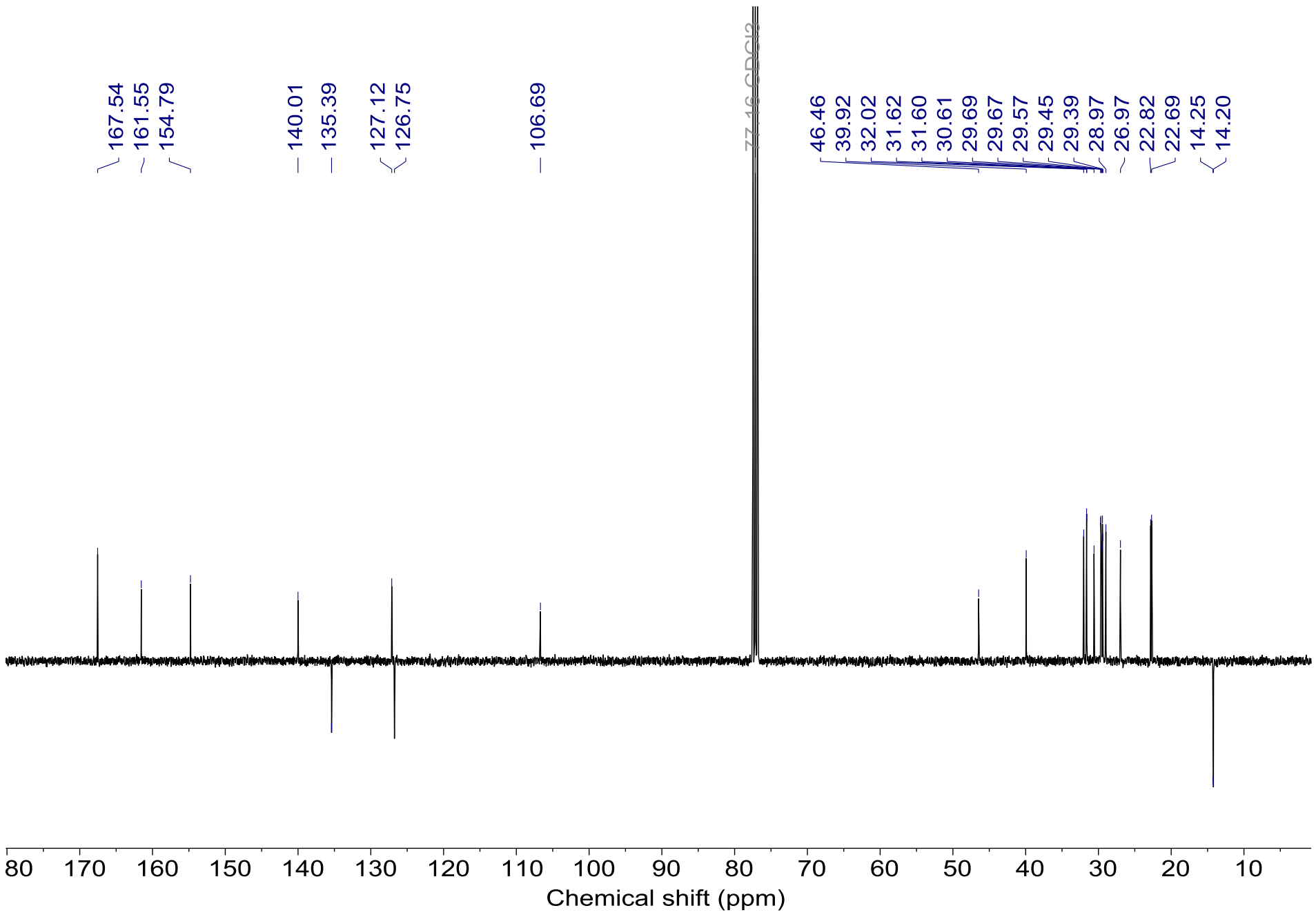

Mass Spectrum HR Report

Analysis Info		Acquisition Date	11/17/2021 2:25:46 PM	
Analysis Name	D:\Data\SMasse\2021\11_November2021\F10963SK.d			
Method	Tune_pos_Mid.m	Operator	BDAL@DE	
Sample Name	HexylDPP-decylacetamide	Instrument	micrOTOF II	8213750.10451
Comment				

Acquisition Parameter					
				Set Corrector Fill	50.9 V
Source Type	ESI	Ion Polarity	Positive	n/a	n/a
n/a	n/a	n/a	n/a	n/a	n/a
Scan Begin	50 m/z	n/a	n/a	Set Reflector	1800.0 V
Scan End	3000 m/z	n/a	n/a	Set Flight Tube	8600.0 V
		n/a	n/a	Set Detector TOF	2008.9 V

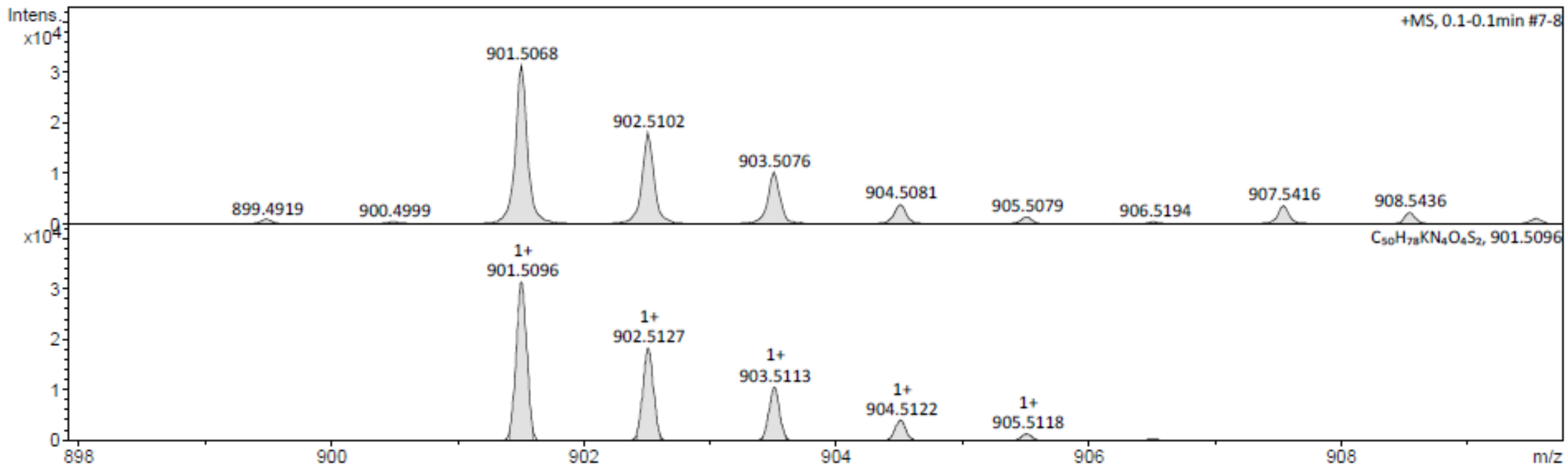

Meas. m/z	#	Ion Formula	m/z	err [ppm]	Mean err [ppm]	rdb	N-Rule	e⁻ Conf	mSigma	Std I	Std Mean m/z	Std I VarNorm	Std m/z Diff	Std Comb Dev
901.506756	1	C50H78KN4O4S2	901.509607	3.2	619.7	13.5	ok	even	7.0	9.4	n.a.	n.a.	n.a.	n.a.

HDPP-4C - di-tert-butyl 4,4'-(3,6-bis(5-hexylthiophen-2-yl)-1,4-dioxopyrrolo[3,4-c]pyrrole-2,5(1H,4H)-diyl)dibutyrate

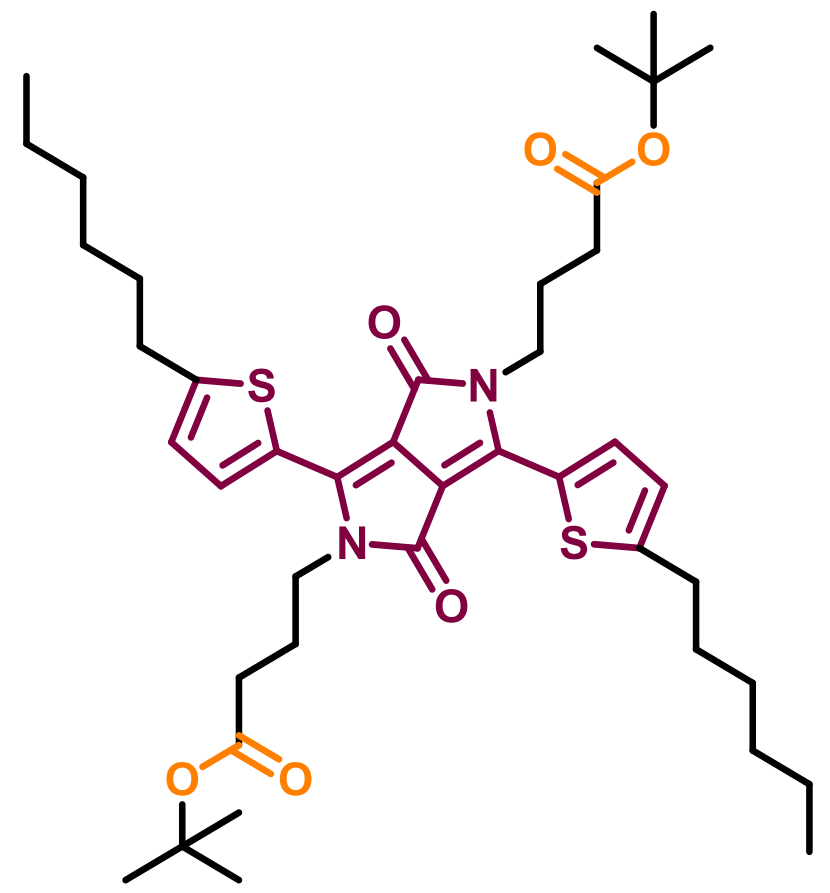

K_2CO_3 (1.1826 g, 8.6 mmol) and tert-Butyl 4-bromobutanoate (1.5 mL, 8.4 mmol) were added to a suspension of **1** (1.0134 g, 2.2 mmol) in DMF (25 mL). The mixture was heated at 120 °C and stirred overnight under argon atmosphere. The reaction was allowed to cool down to room temperature, the solvent was evaporated under reduced pressure. The residue was dissolved in $CHCl_3$ (70 mL) and washed with water (4x 70 mL). The organic phase was dried over $MgSO_4$, filtered and evaporated to dryness under vacuum. The crude product was purified by column chromatography using toluene-ethyl acetate (0% up to 0.5%) as eluent. The desired product was obtained as a dark violet solid (0.2323 g, 13%).

^{1}H NMR (400 MHz, $CDCl_3$) δ 8.72 (d, *J* = 3.9 Hz, 2H , Ar*H*), 6.96 (d, *J* = 4.0 Hz, 2H, Ar*H*), 4.11 (t, *J* = 7.3 Hz, 4H, N-CH_2-), 2.89 (t, *J* = 7.7 Hz, 4H, Thiophene-CH_2-), 2.35 (t, *J* = 7.8 Hz, 4H, -CH_2-CONH-), 2.08 – 1.99 (m, 4H, -CH_2-), 1.73 (p, *J* = 7.6 Hz, 4H, -CH_2-), 1.43 (s, 18H, OC-CH_3), 1.39 – 1.27 (m, 12H, -CH_2-), 0.92 – 0.87 (m, 6H, -CH_3).

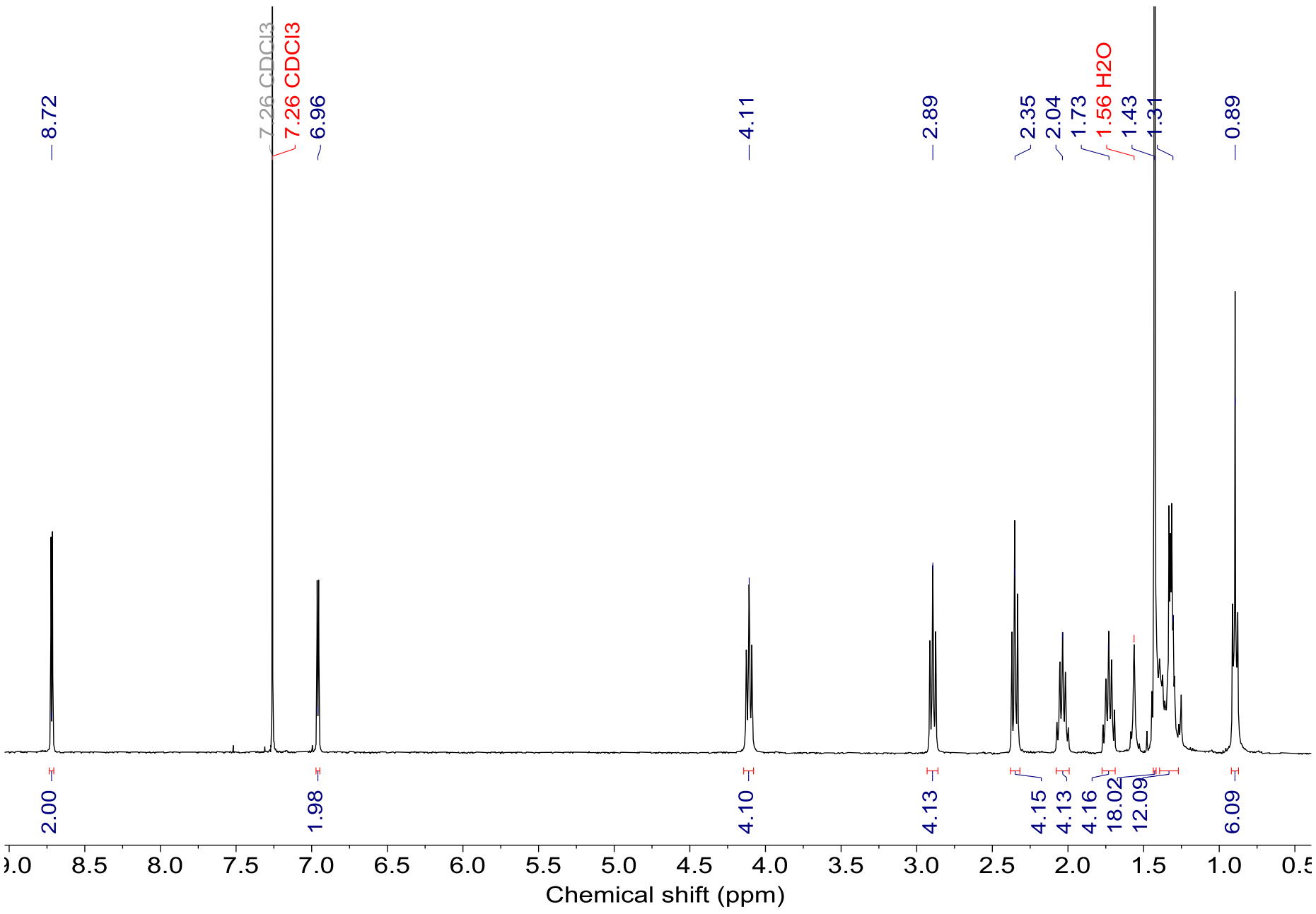

HDPP-4C-acid - 4,4'-(3,6-bis(5-hexylthiophen-2-yl)-1,4-dioxopyrrolo[3,4-c]pyrrole-2,5(1H,4H)-diyl)dibutyric acid

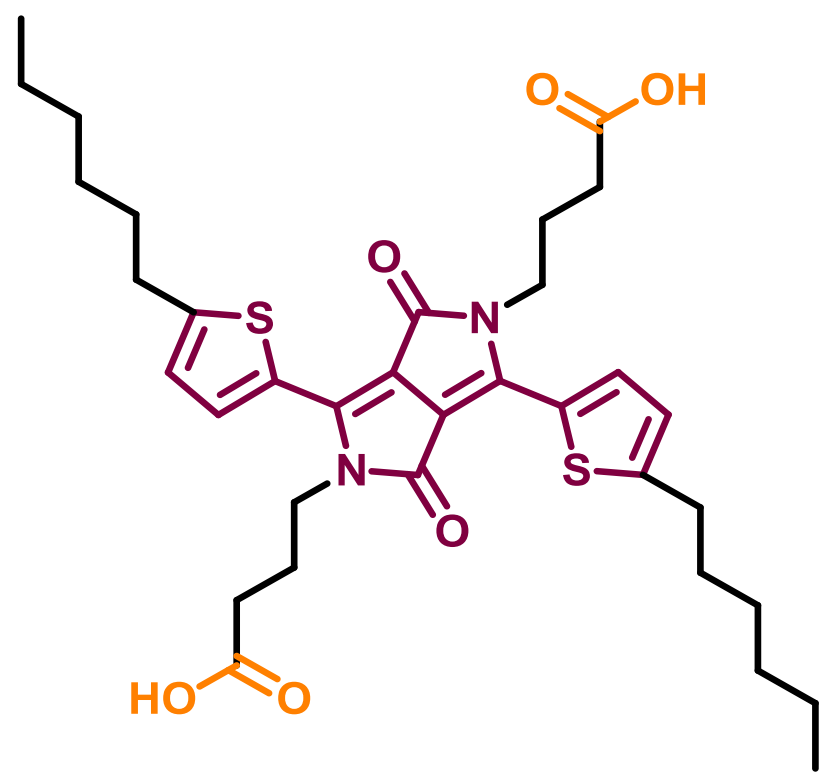

HDPP-4C (0.2323 g, 0.36 mmol) was dissolved in dichloromethane (6 mL) at room temperature. Then, trifluoroacetic acid was added (0.7 mL) in fractions during 2 h. The reaction was stopped after 6 h. Next, ethyl ether (80 mL) was added to the reaction. The flask was put in an ice bath and the mixture was stirred for 20 minutes. After filtrating the obtained solid was washed with ethyl ether several times. Following drying under reduced pressure the target compound was afforded as a dark violet solid (0.1752 g, 89%) in sufficient purity.

^{1}H NMR (400 MHz, DMSO) δ 12.15 (s, 2H, -O*H*), 8.66 (d, *J* = 3.9 Hz, 2H, Ar*H*), 7.13 (d, *J* = 3.9 Hz, 2H, Ar*H*), 4.00 (t, *J* = 7.2 Hz, 4H, N-CH_2-), 2.91 (t, *J* = 7.6 Hz, 4H , Th-CH_2-), 2.30 (t, *J* = 7.3 Hz,

4H, -CH_2-CONH), 1.85 (p, J = 7.4 Hz, 4H, -CH_2-), 1.68 (p, J = 7.5 Hz, 4H, -CH_2-), 1.38 – 1.26 (m, 12H, -CH_2-), 0.89 – 0.83 (m, 6H, -CH_3).

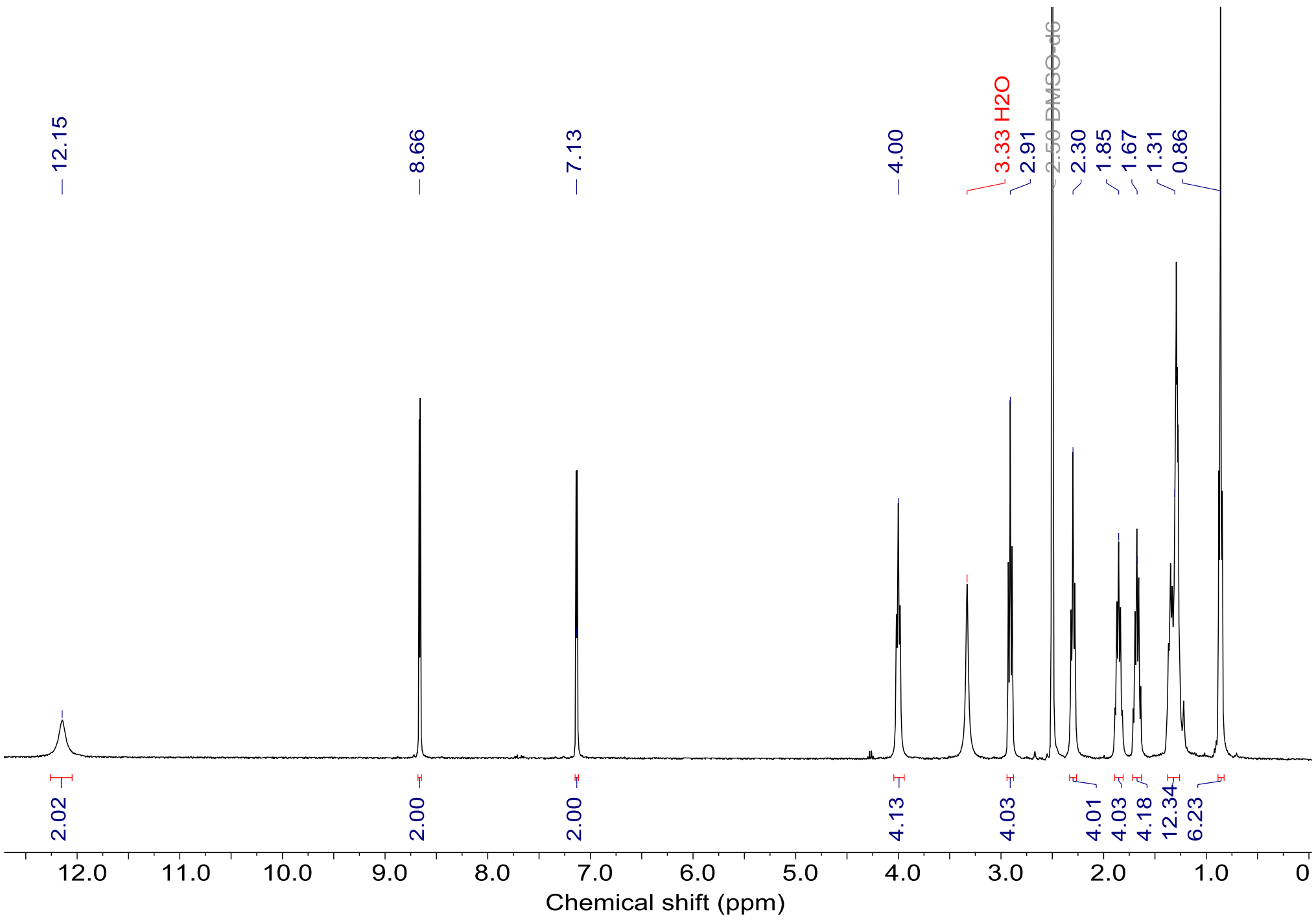

HDPPBA-4C - 4,4'-(3,6-bis(5-hexylthiophen-2-yl)-1,4-dioxopyrrolo[3,4-c]pyrrole-2,5(1H,4H)-diyl)bis(N-octylbutanamide)

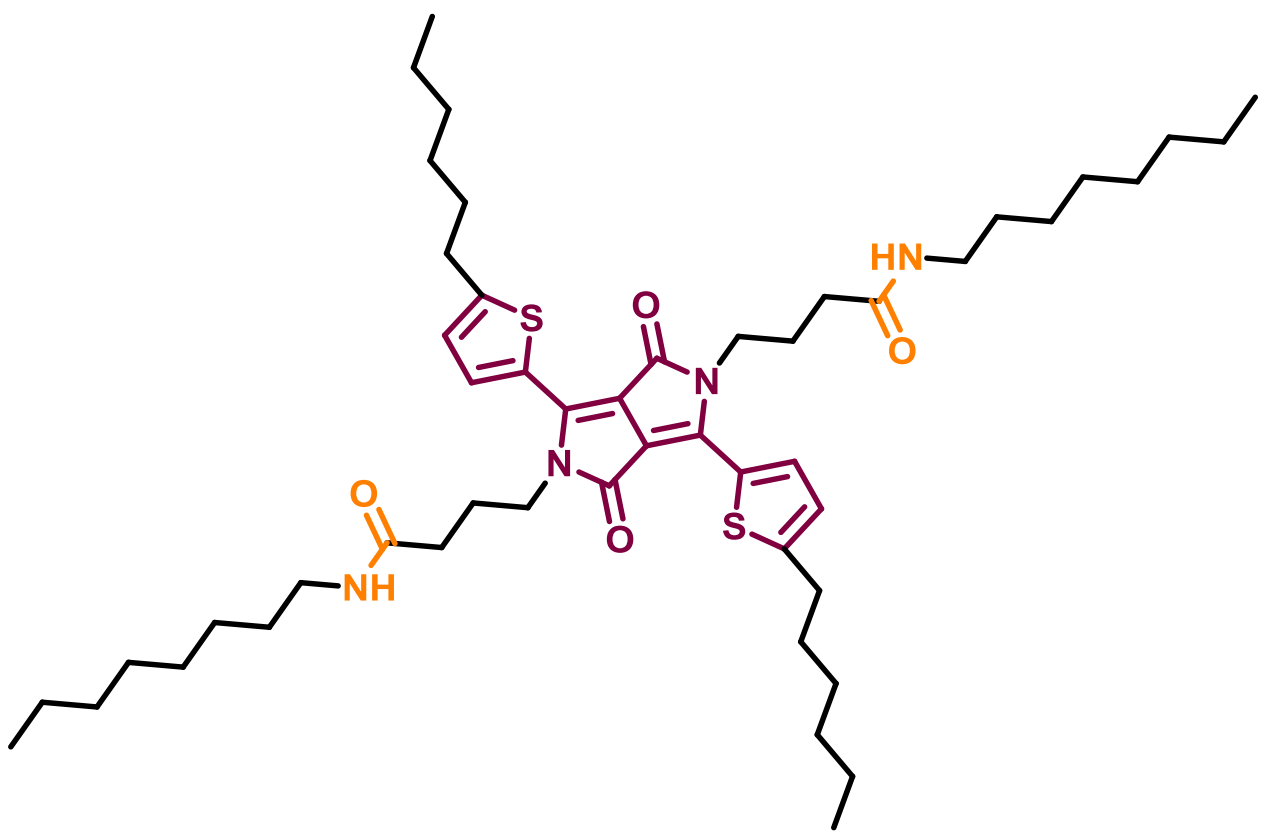

HDPP-4C-acid (0.1706 g, 0.2 mmol), HBTU (0.4058 g, 1.0 mmol) and octylamine (0.2 mL, 1.2 mmol) were dissolved in dry DMF (5 mL) under argon while stirring. After 5 min DIPEA (0.3 mL, 1.7 mmol) was added to the reaction mixture and it was stirred under argon atmosphere overnight at

50 °C. After 17 h, the starting material was still visible by TLC therefore 4 more equivalents of octylamine, HBTU and DIPEA each were added to the reaction. After 5 h more the reaction was stopped. DMF was removed under vacuum at 70 °C. Then, the remaining solid was dissolved in the minimum volume of hot $CHCl_3$ and poured in diethyl ether (100 mL). The resulting solid was washed with diethyl ether and then recrystallized again in a $CHCl_3$-methanol mixture to obtain the desired pure product as dark purple solid (0.2054 g, 89%).

^{1}H NMR (400 MHz, $CDCl_3$) δ 8.74 (d, *J* = 3.9 Hz, 2H, Ar*H*), 6.97 (d, *J* = 3.9 Hz, 2H, Ar*H*), 6.28 (s, 2H, N*H*), 4.14 (t, *J* = 6.3 Hz, 4H, N-CH_2-), 3.24 (q, *J* = 5.8 Hz, 4H, -CONH-CH_2), 2.90 (t, *J* = 7.7 Hz, 4H, Thiophene-CH_2), 2.27 (t, *J* = 7.0 Hz, 4H, -CH_2-CONH), 2.11 (p, *J* = 6.9 Hz, 4H, -CH_2-), 1.73 (p, 4H, -CH_2-), 1.55 – 1.47 (m, 4H, -CH_2-), 1.43 – 1.36 (m, 4H, -CH_2-), 1.35 – 1.23 (m, 28H, -CH_2-), 0.92 – 0.84 (m, 12H, -CH_3).

^{13}C NMR (101 MHz, $CDCl_3$) δ 172.2, 162 , 153.8, 140, 136, 127.2, 126.4, 106.9, 41.2, 39.8, 33.9, 31.97, 31.6, 31.6, 30.52, 29.7, 29.4, 29.4, 28.9, 27.1, 26.7, 22.7, 14.2.

Theoretical $[M+H]^+$: 862.5464, HR-MS (ESI-TOF): 863.5504 $[M+H]^+$.

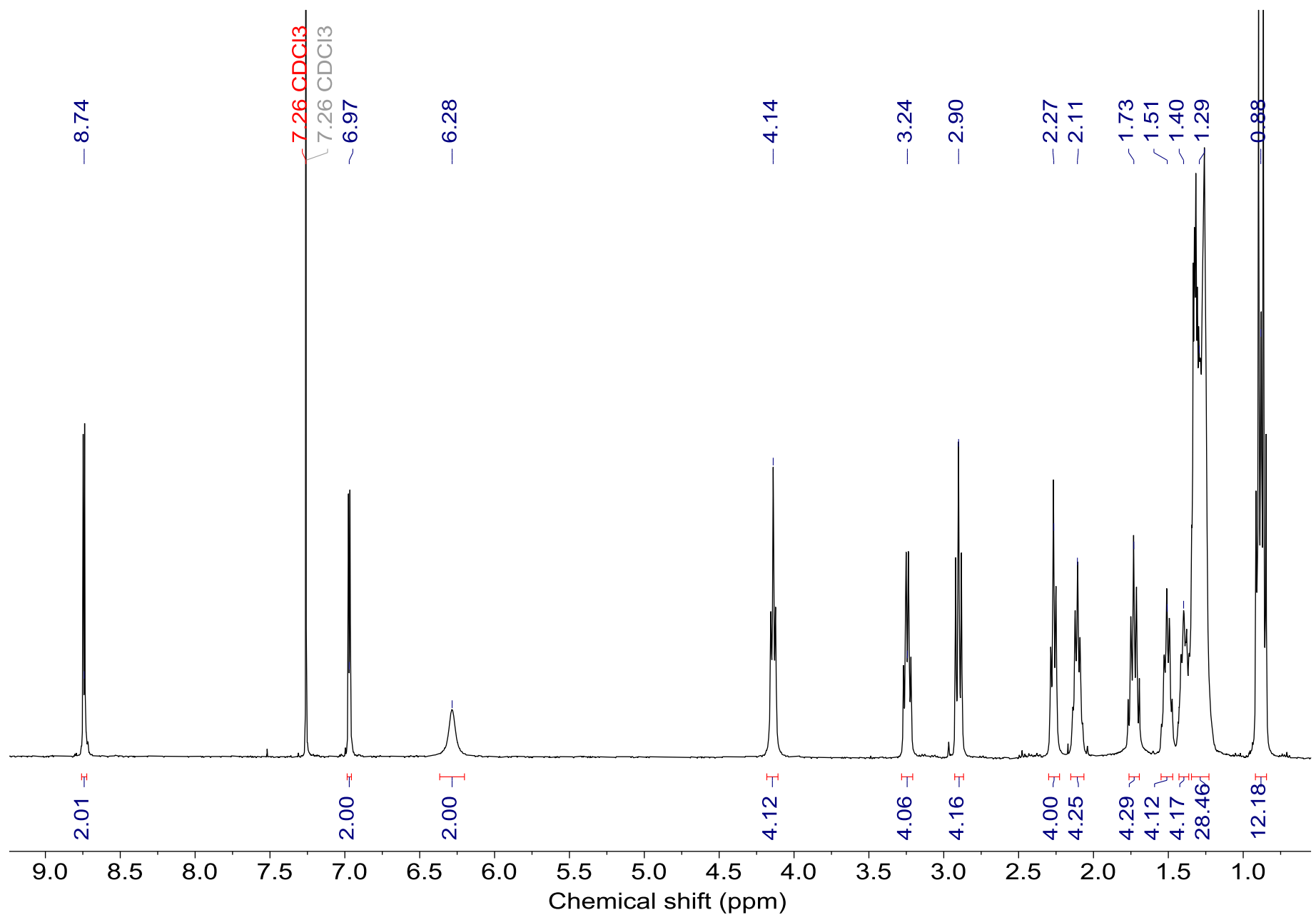

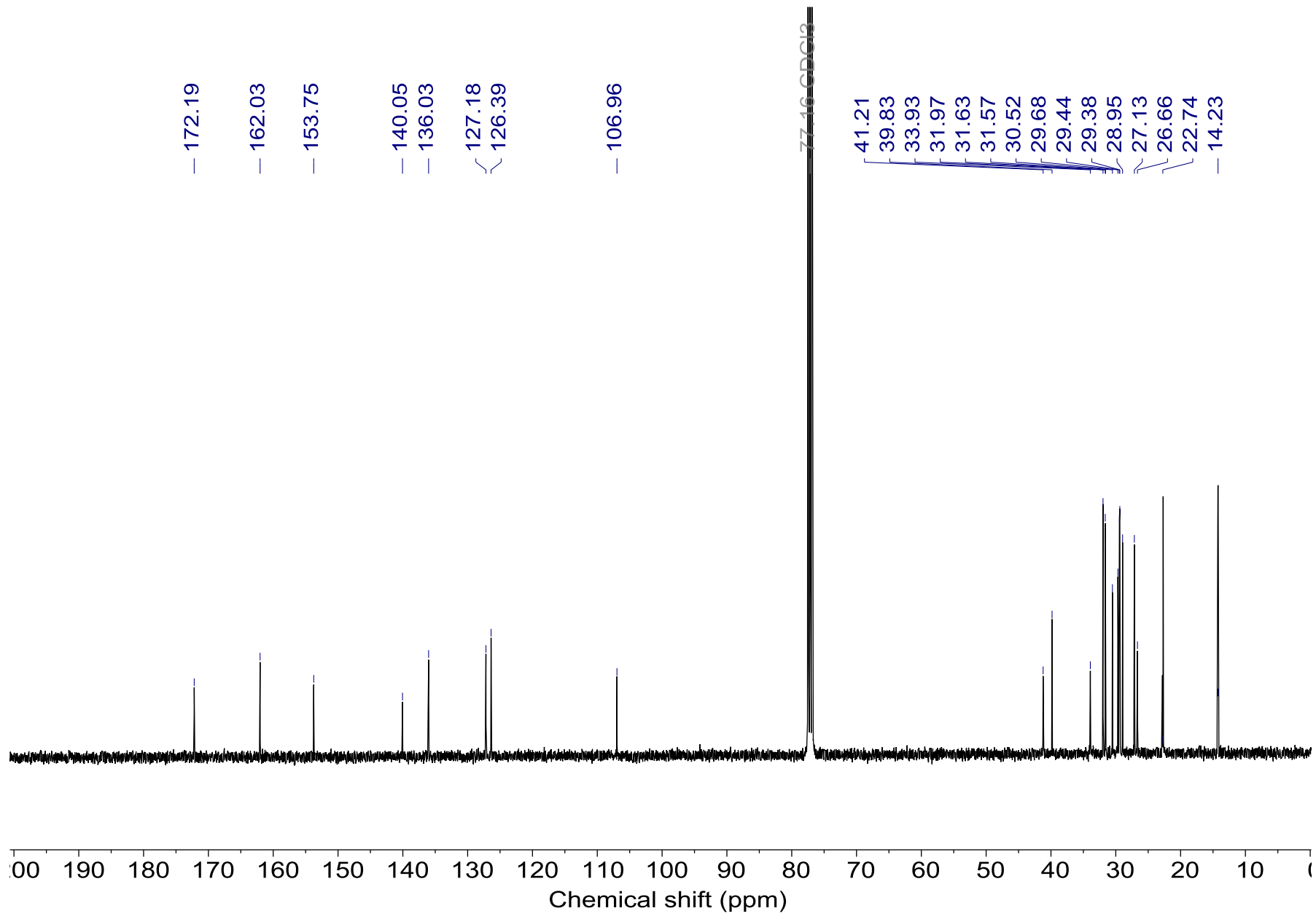

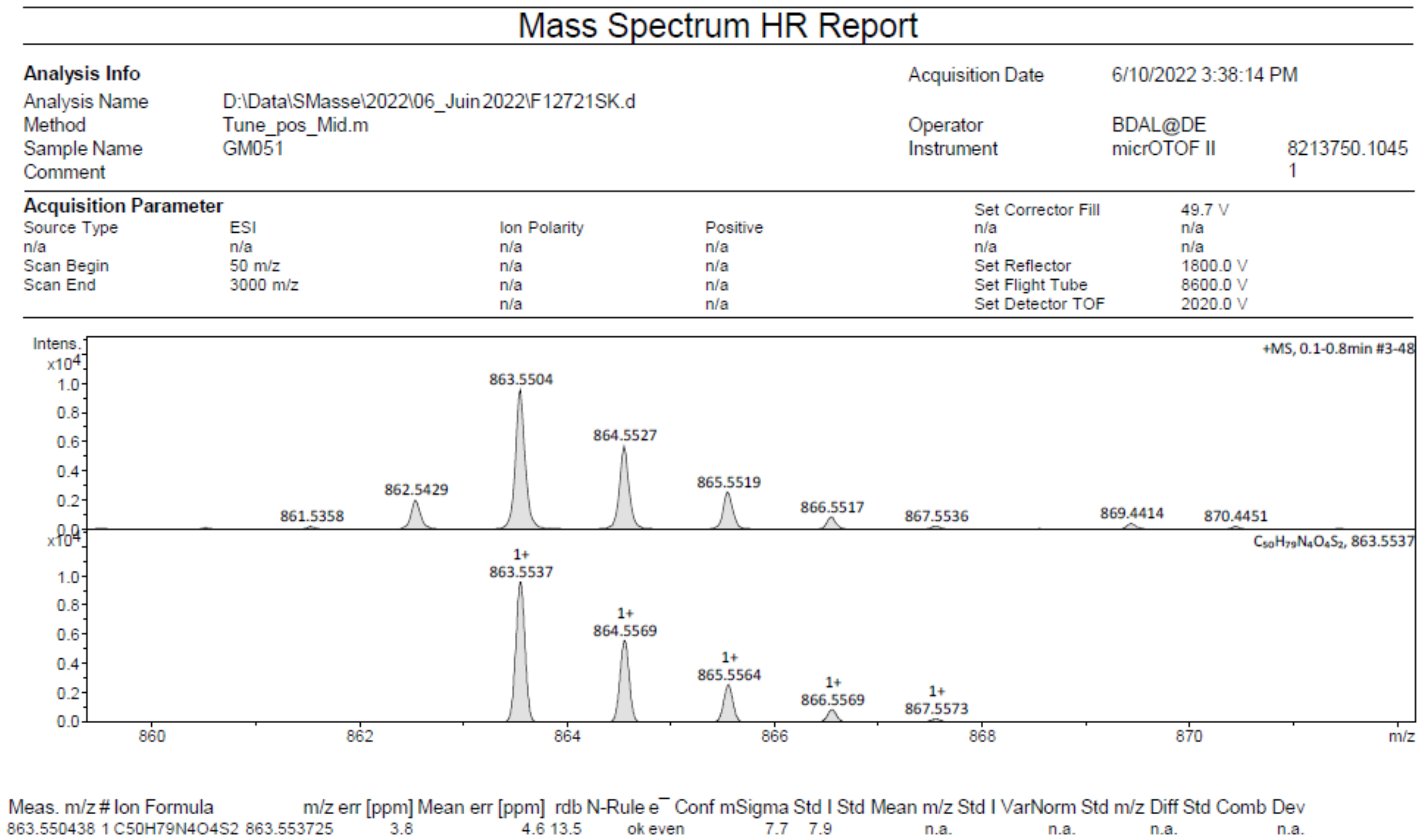

DFT and TD-DFT calculations

Theoretical calculations for **HDPPH**, **HDPPBA-2C**, **HDPPBA-4C** and **HDPPBA-6C** derivates were carried out in the frame of density functional theory (DFT), using the B3LYP[1,2] functional and the 6-31G** basis set[3,4] as implemented in the Gaussian 16 program. Geometry optimizations were

performed without any symmetry constrains. Based on the resulting ground-state geometries, harmonic vibrational frequencies and TD-DFT[5,6] were calculated at the same theoretical level.

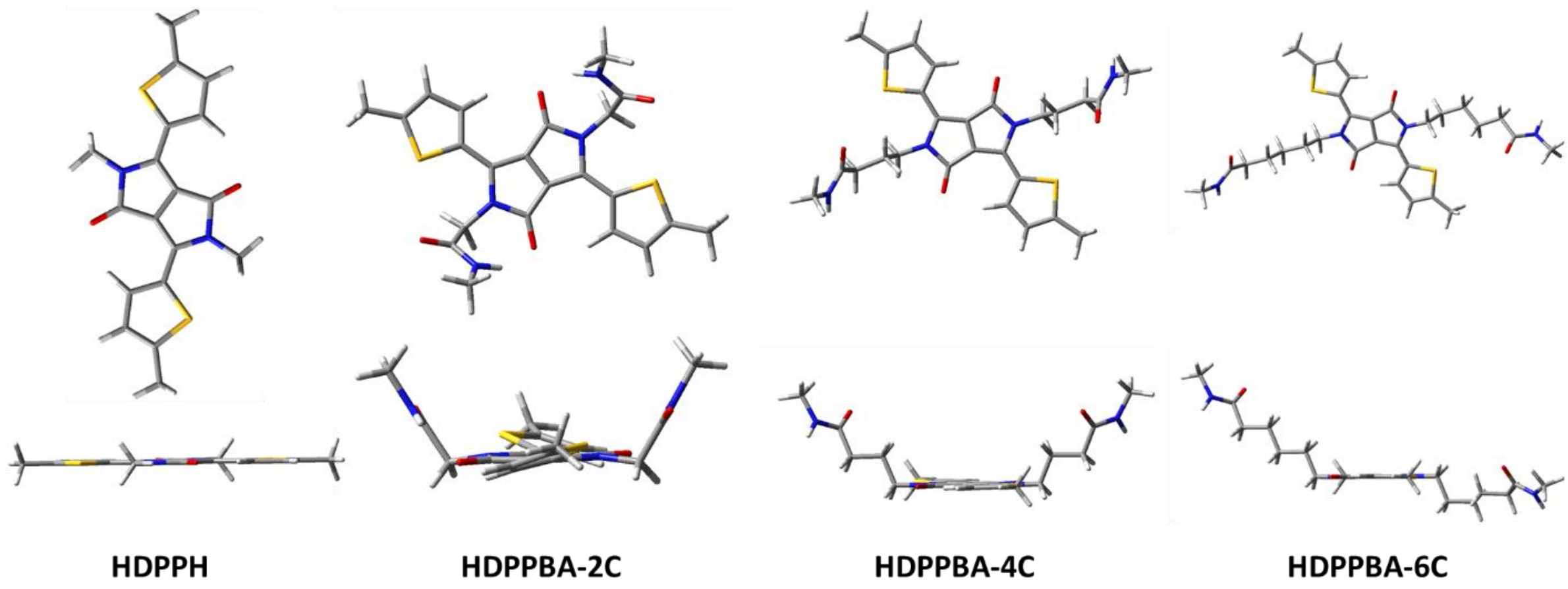

Figure S1. DFT//B3LYP/6-31G** optimized molecular structures of **HDPPH**, **HDPPBA-2C**, **HDPPBA-4C** and **HDPPBA-6C**.

To evaluate the possible formation of an intramolecular hydrogen bond, we have systematically modelled the alternative conformations of **HDPPBA-2C** (denoted as conf-2, conf-3 and conf-4) using DFT optimization at B3LYP/6-31G** level of theory. The optimized structures and their relative energies are presented below. As illustrated in Figure S2, the alternative conformations evaluated (**HDPPBA-2C-conf-2**, **HDPPBA-2C-conf-3** and **HDPPBA-2C-conf-4**) are all less stable than the original global minimum structure (**HDPPBA-2C**), sitting between 0.4 kcal/mol and 3 kcal/mol higher in energy. Given these energy differences, only **HDPPBA-2C-conf-2** is energetically accessible enough to be expected to contribute appreciably to the bulk material. Importantly, **HDPPBA-2C** and **HDPPBA-2C-conf-2** exhibit comparable backbone distortions and intramolecular hydrogen-bonding patterns. Consequently, the packing model and thin-film analysis originally derived from the global minimum structures remain representative of the physical systems.

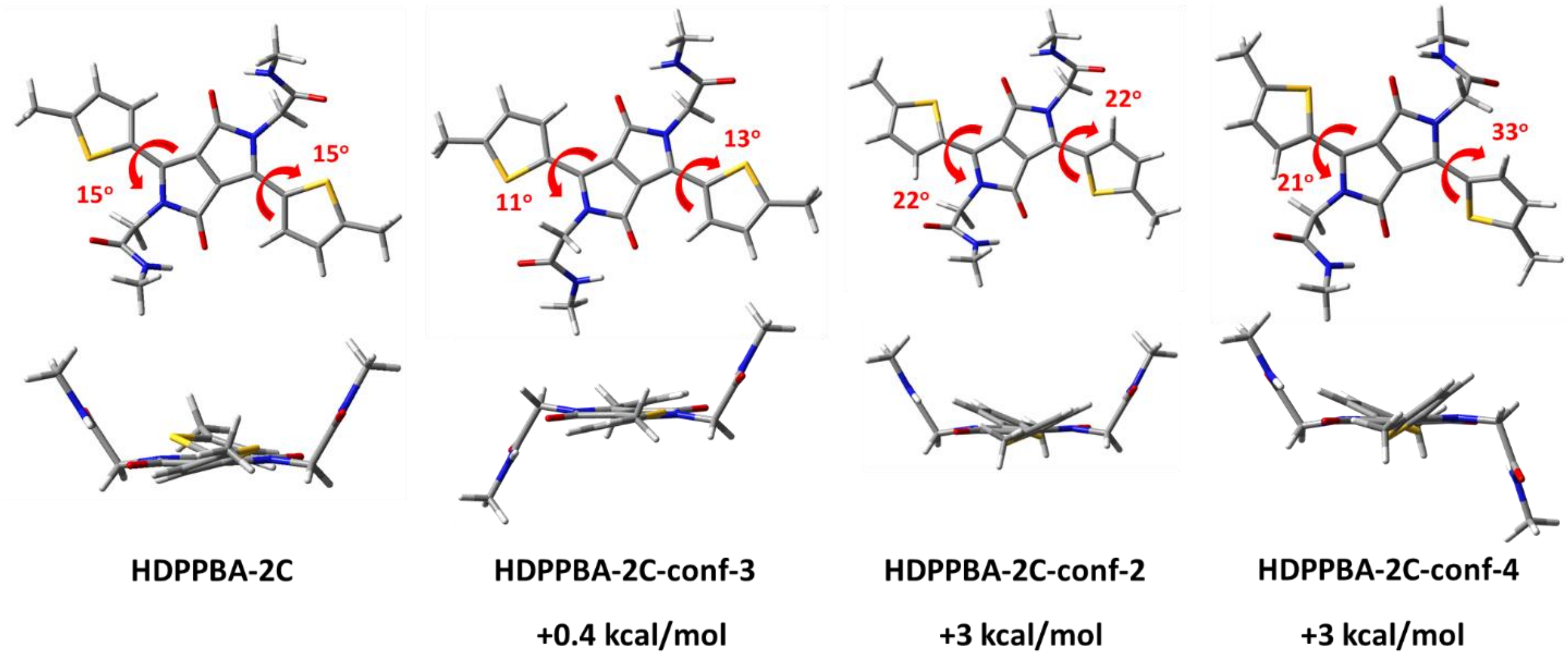

Figure S2. Optimized geometries and relative DFT energies at B3LYP/6-31G** level for the global minimum and alternative conformations (conf-2, conf-3 and conf-4) of **HDPPBA-2C**.

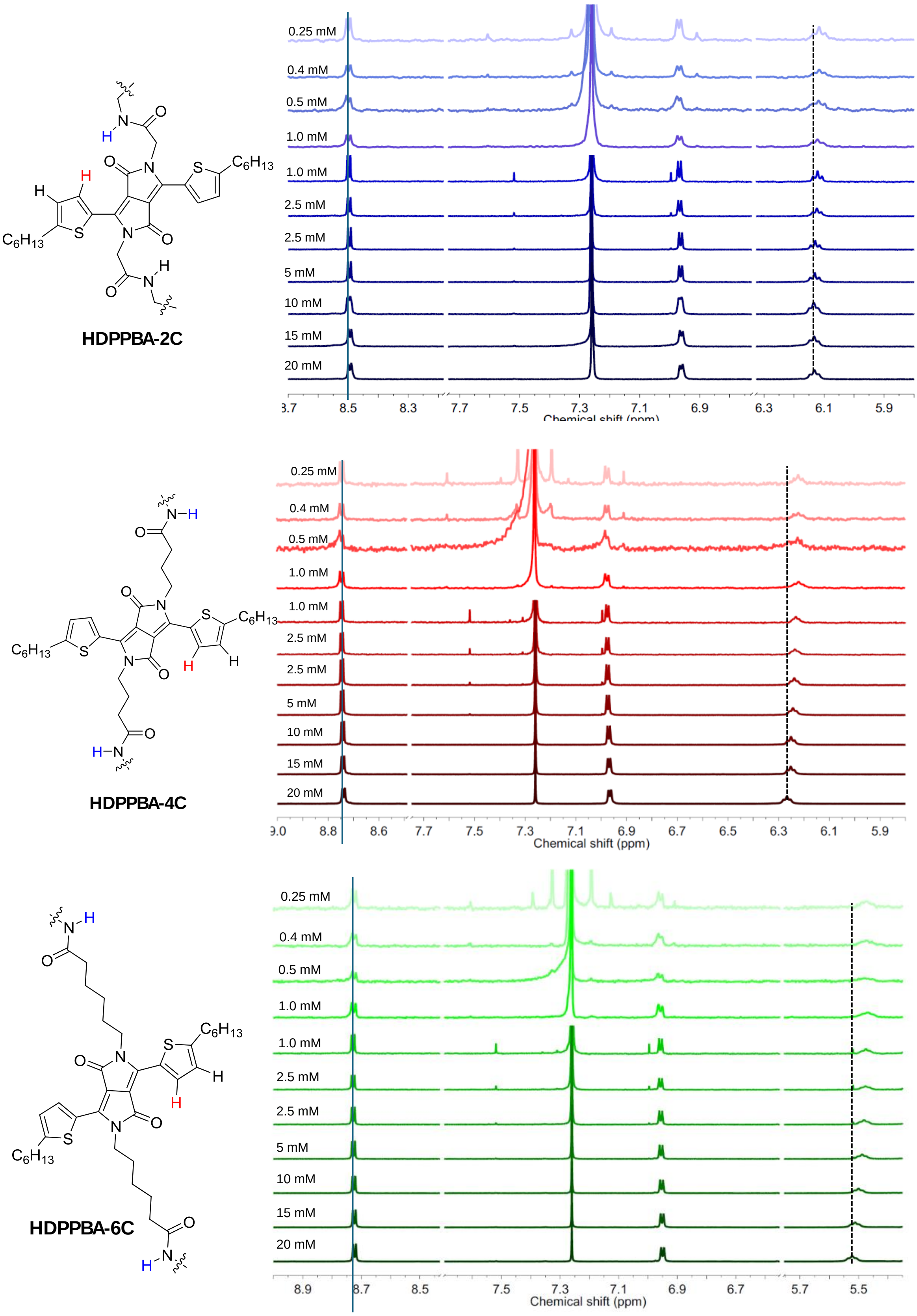

Figure S3. Partial concentration-dependent (20 mM to 0.25 mM) ^{1}H-NMR spectra in $CDCl_3$ showing the aromatic and amide protons for **HDPPBA-2C**, **HDPPBA-4C** and **HDPPBA-6C**.

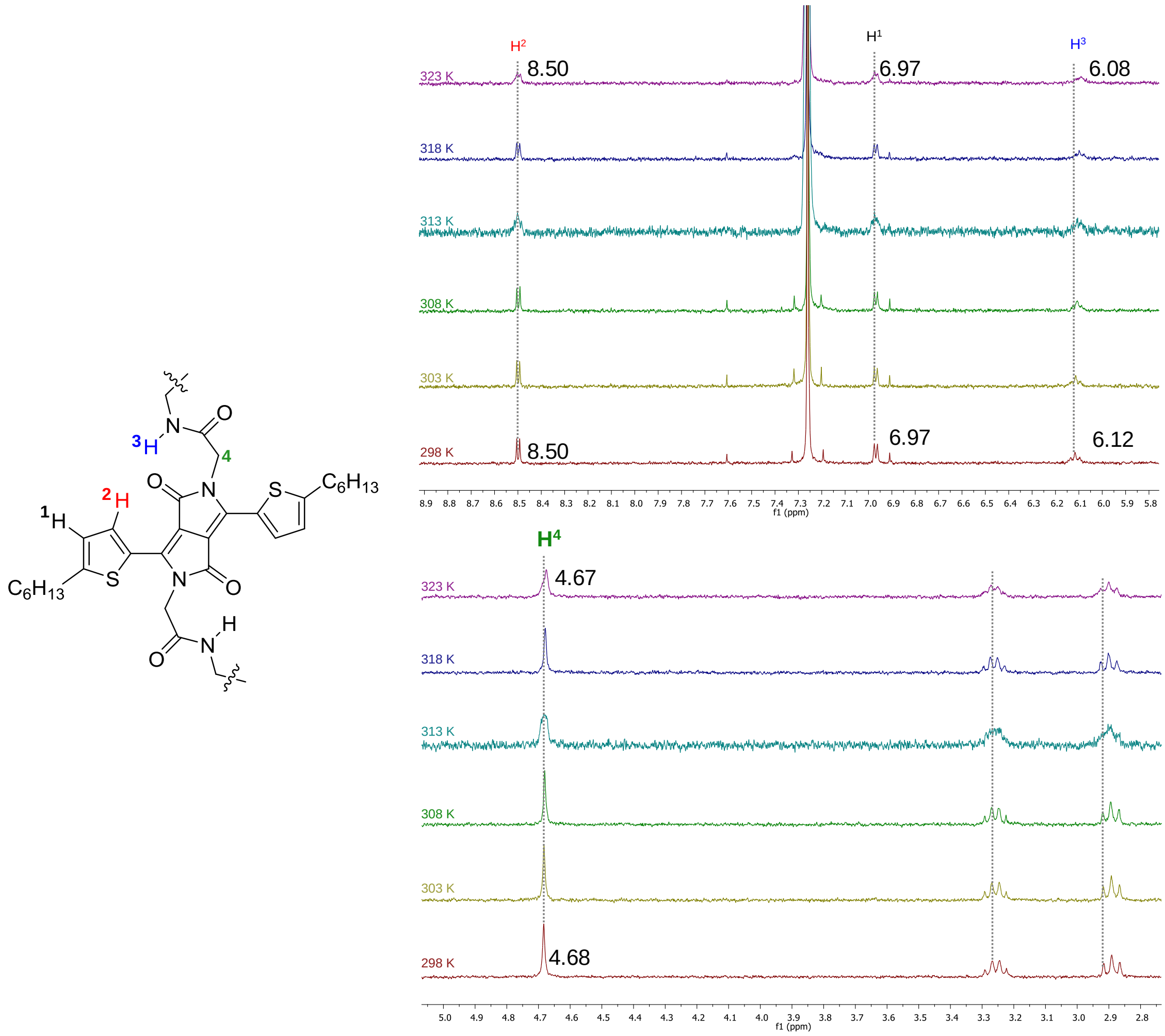

Figure S4. Partial ^{1}H-NMR spectra of **HDPPBA-2C** at 0.25 mM at variable temperature showing the aromatic protons (1 and 2), the amide protons (3) and some of the aliphatic signals (4).

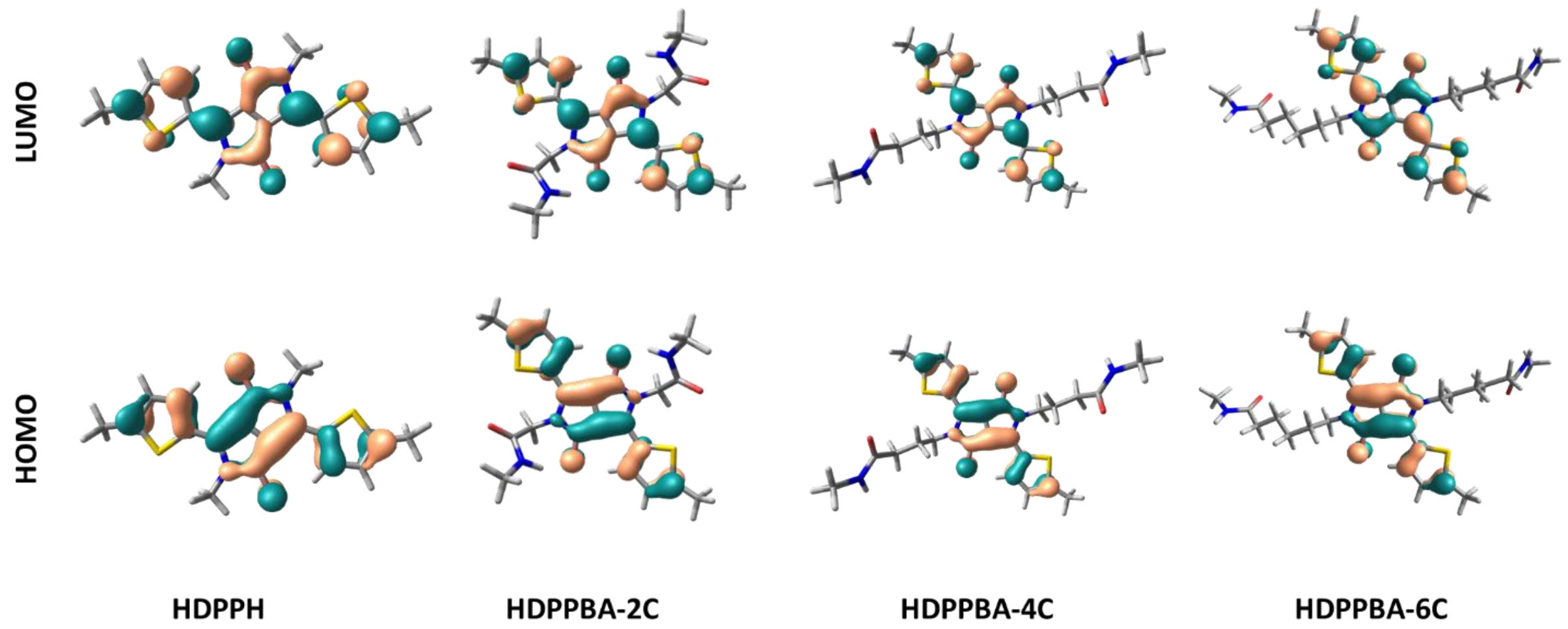

Figure S5. Molecular orbital topologies of HOMO and LUMO orbitals of **HDPPH**, **HDPPBA-2C**, **HDPPBA-4C** and **HDPPBA-6C** (isovalue of 0.035 a.u).

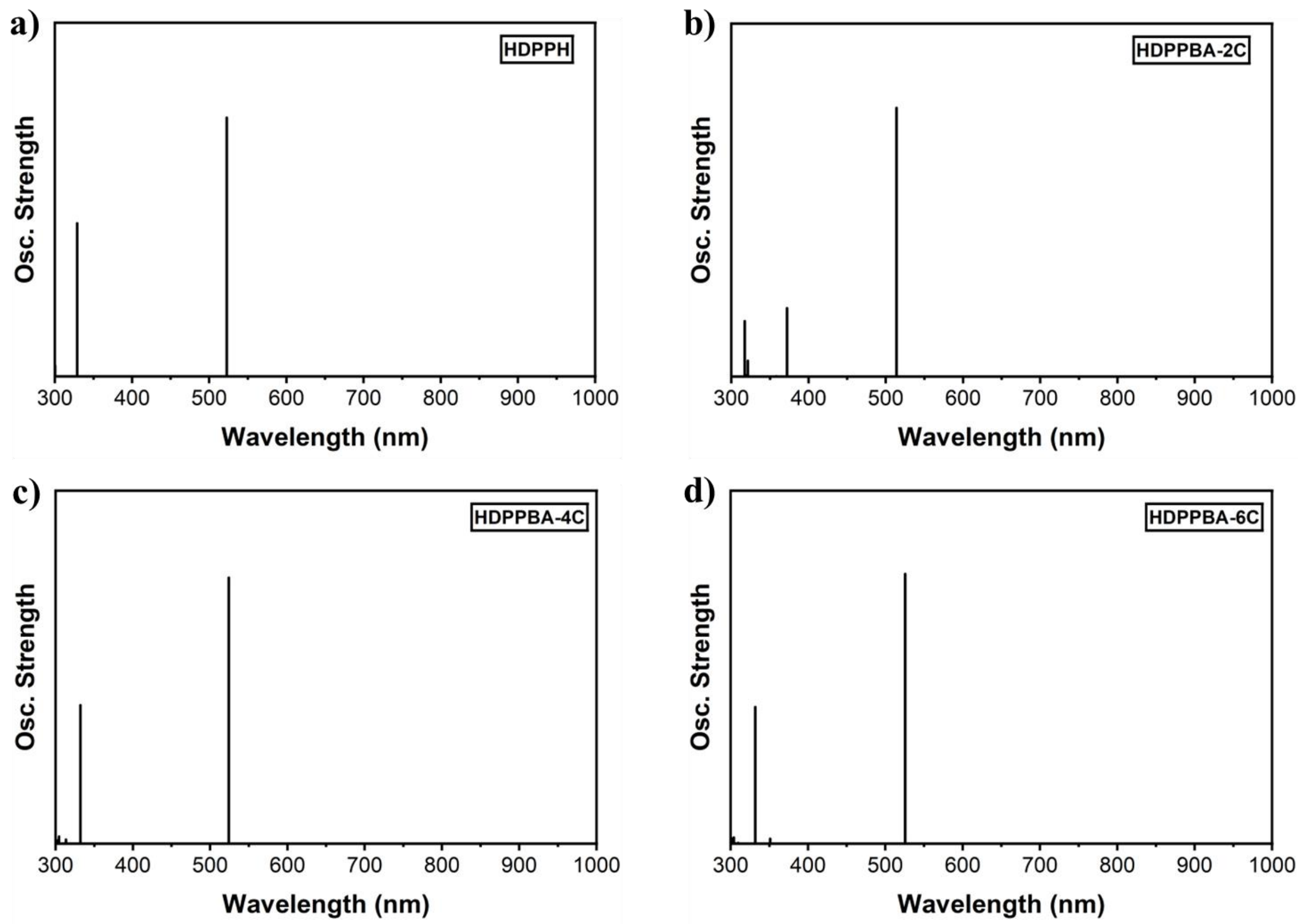

Figure S6. TD-DFT calculated vertical transition energies of a) **HDPPH**, b) **HDPPBA-2C**, c) **HDPPBA-4C** and d**) HDPPBA-6C**.

Table S1. Electronic transitions obtained by TD-DFT B3LYP/6-31G** level of theory for **HDPPH**, **HDPPBA-2C**, **HDPPBA-4C** and **HDPPBA-6C**.

Compound	Description	λ_{max}	f
**HDPPH**	H→L (100%)	523 nm	0.59
**HDPPBA-2C**	H→L (100%)	514 nm	0.61
**HDPPBA-4C**	H→L (100%)	524 nm	0.60
**HDPPBA-6C**	H→L (100%)	525 nm	0.61

Vibrational Study

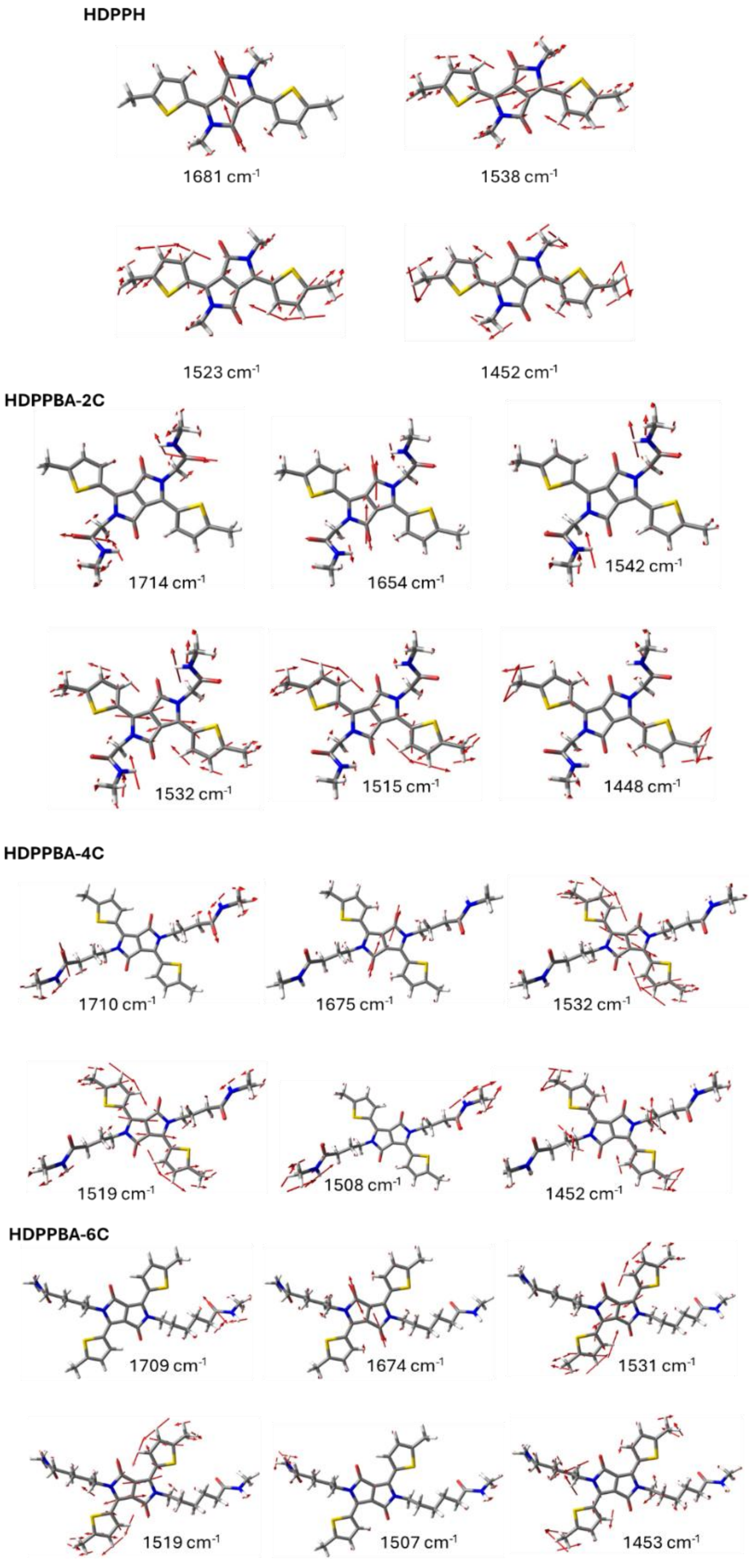

Figure S7. B3LYP/6-31G** IR predicted eigenvectors for **HDPPH**, **HDPPBA-2C**, **HDPPBA-4C** and **HDPPBA-6C.**

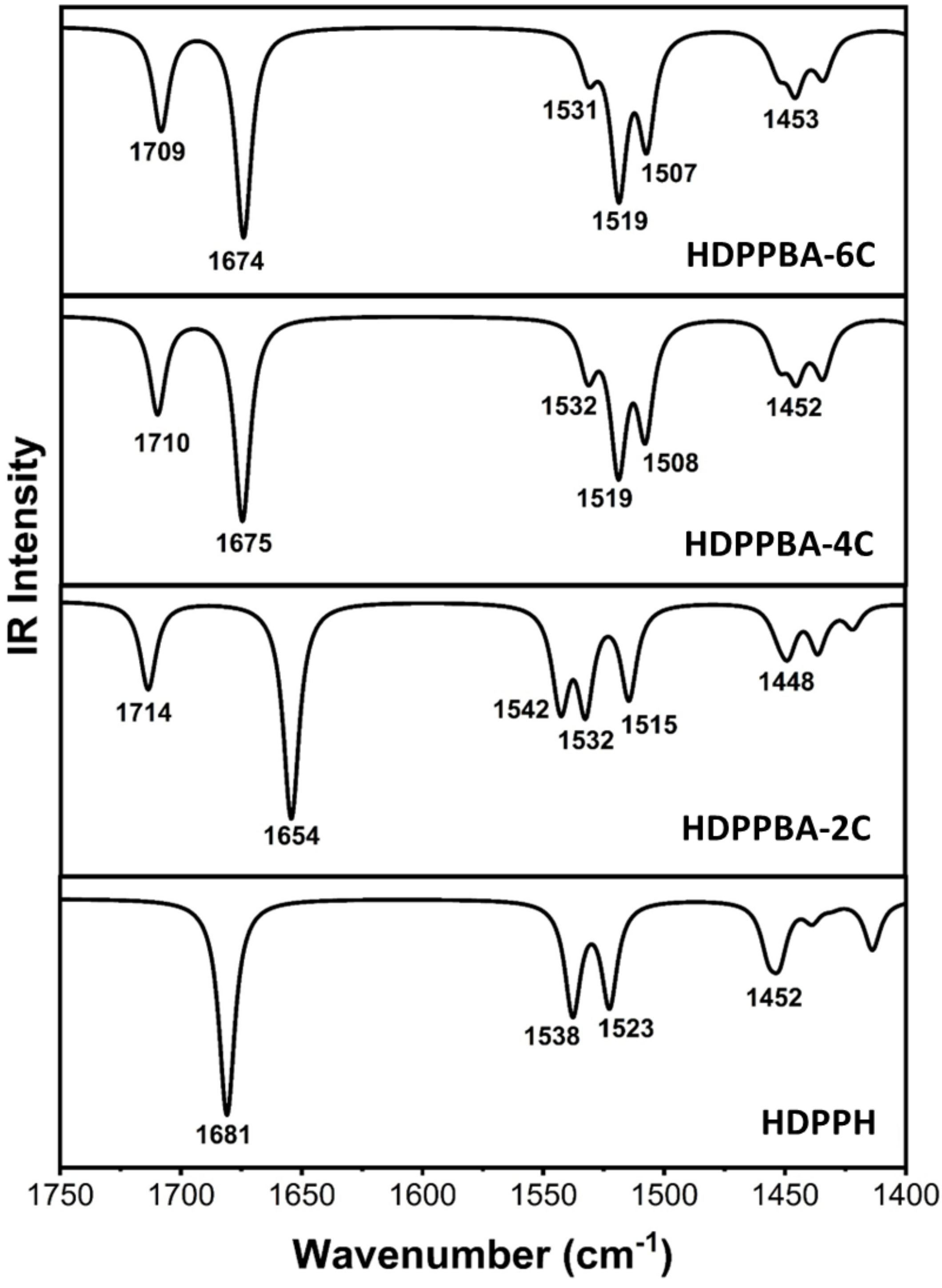

Figure S8. B3LYP/6-31G** IR theoretical spectra (f=0.96) for **HDPPH**, **HDPPBA-2C**, **HDPPBA-4C** and **HDPPBA-6C**.

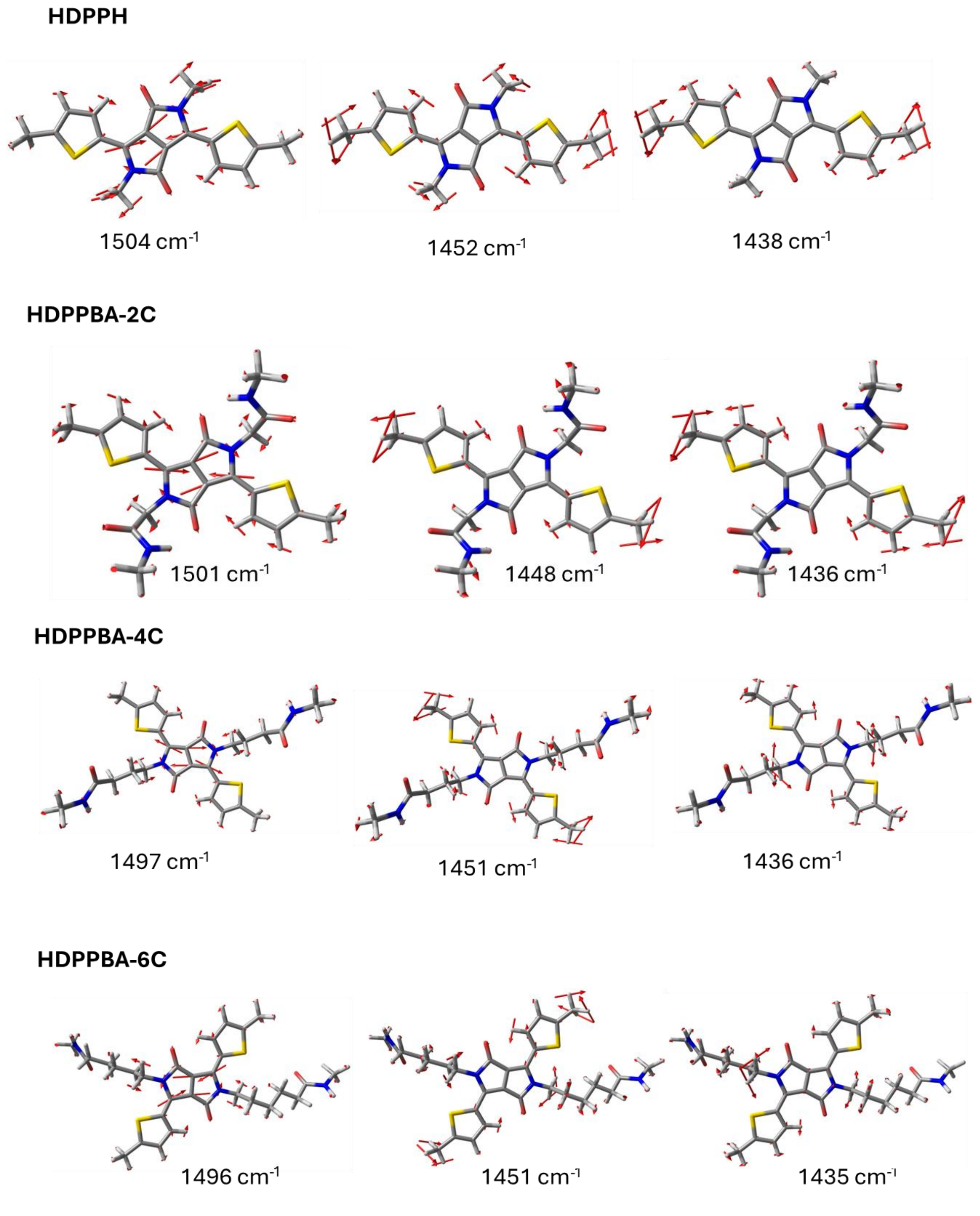

Figure S9. B3LYP/6-31G** Raman predicted eigenvectors for **HDPPH**, **HDPPBA-2C**, **HDPPBA-4C** and **HDPPBA-6C.**

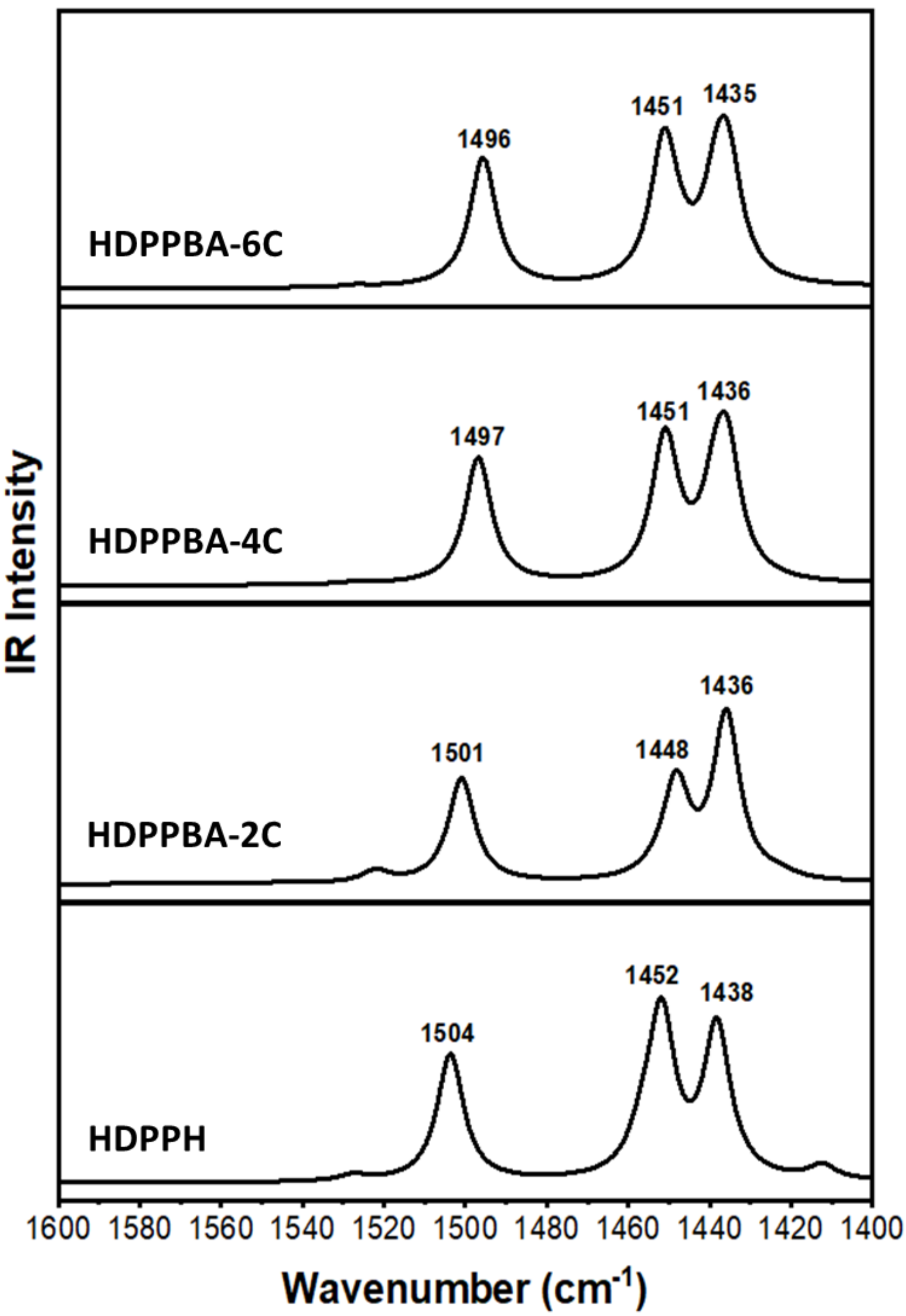

Figure S10. B3LYP/6-31G** Raman theoretical spectra (f=0.96) for **HDPPH**, **HDPPBA-2C**, **HDPPBA-4C** and **HDPPBA-6C**.

Electrochemical data

Table S2. Experimental frontier orbital energy levels for **HDPPH**, **HDPPBA-2C**, **HDPPBA-4C** and **HDPPBA-6C** estimated from cyclic voltammetry measurements in solution and estimated optical band gaps.

	Eox (onset,V)	Ered (onset, V)	HOMO (eV)	LUMO (eV)	ΔE
**HDPPH**	0.72	-0.85	-5.1	-3.37	1.73
**HDPPBA-2C**	0.76	-0.83	-5.14	-3.39	1.75
**HDPPBA-4C**	0.72	-0.88	-5.1	-3.34	1.76
**HDPPBA-6C**	0.71	-0.85	-5.09	-3.37	1.72

Spectroelectrochemistry

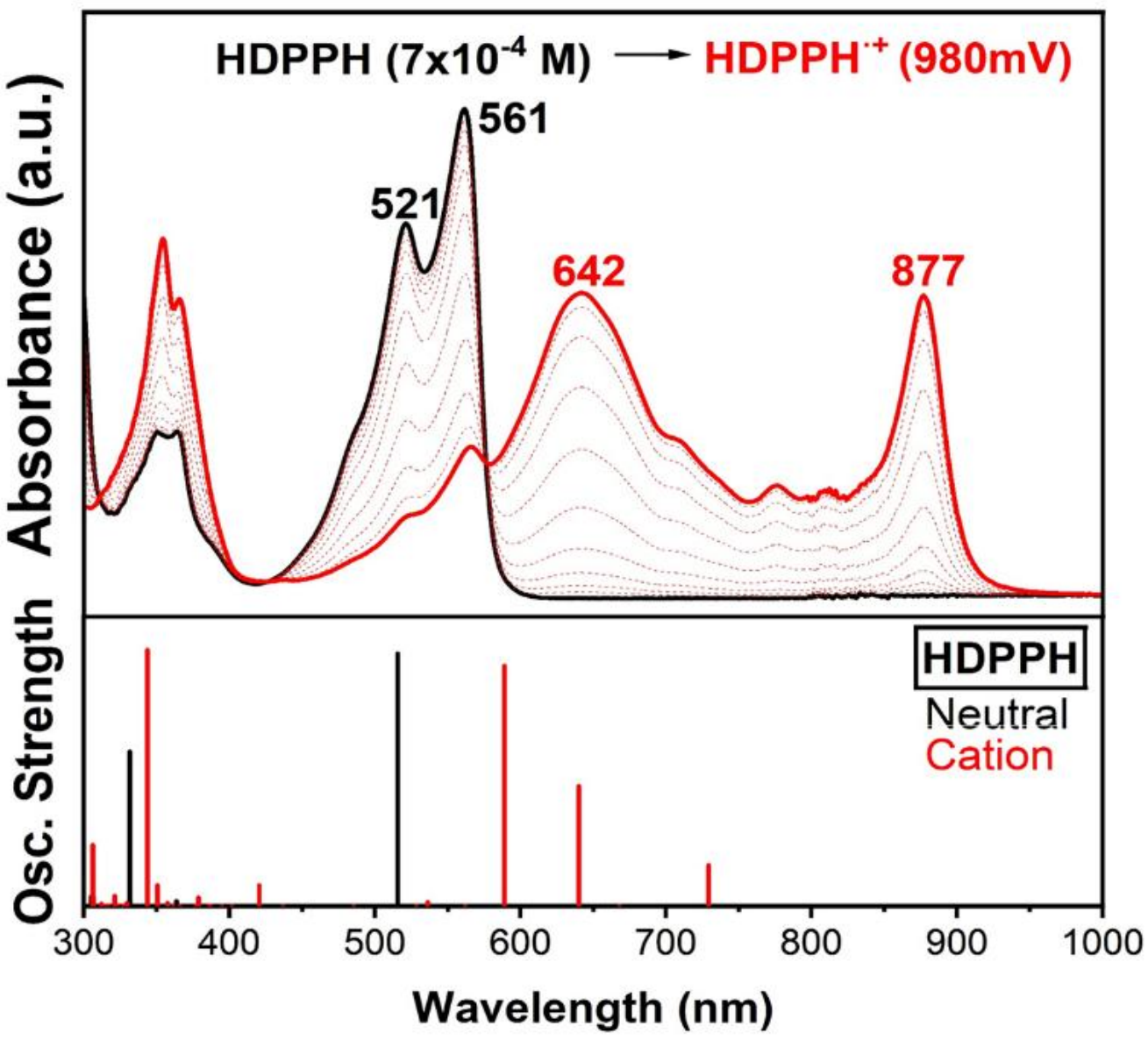

Figure S11. a) UV-Vis-NIR absorption spectra of **HDPPH** electrochemically oxidized by progressive increase of the oxidation potentials; b) TDDFT-B3LYP/6-31G**-calculated vertical transition energies.

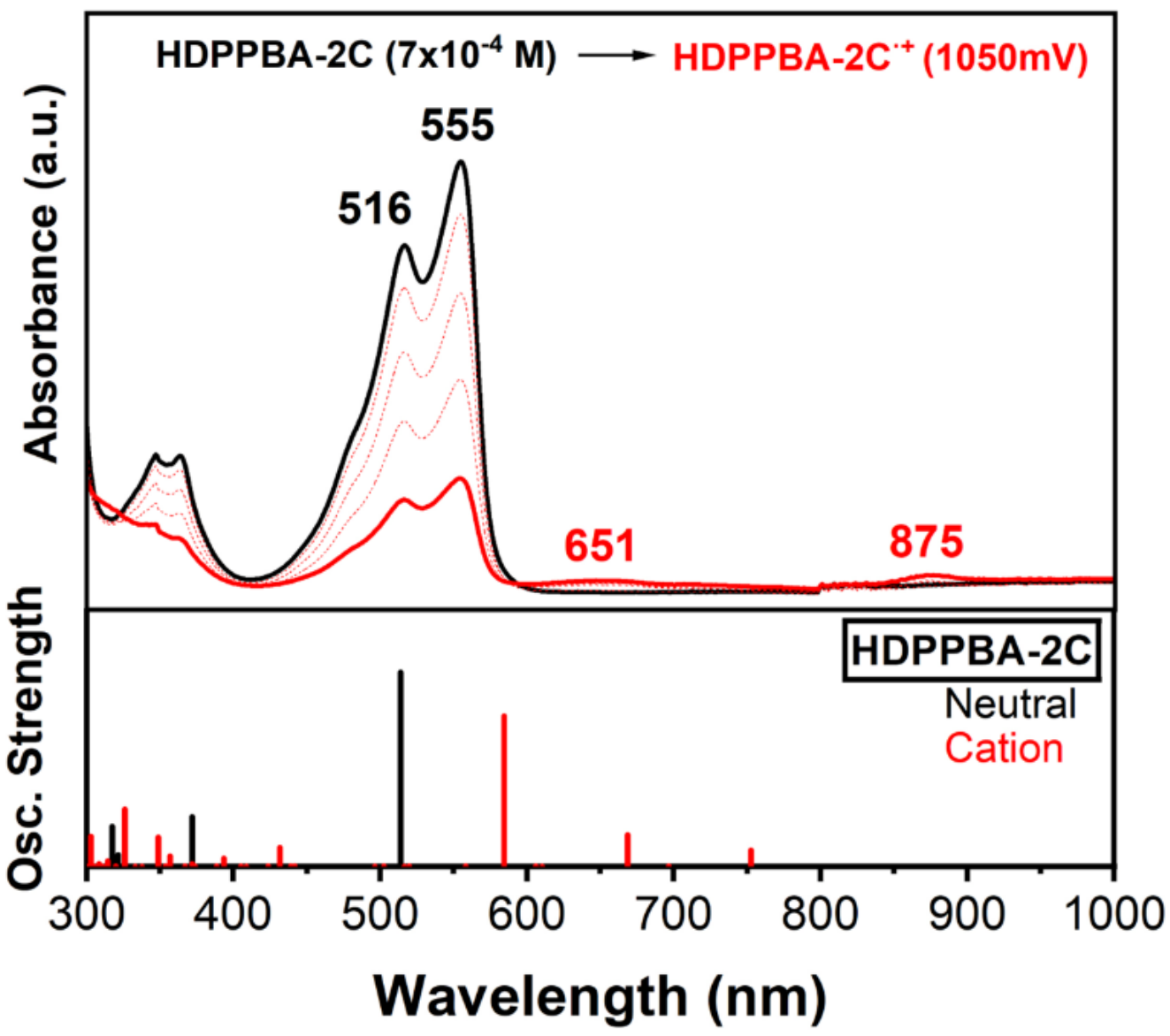

Figure S12. a) UV-Vis-NIR absorption spectra of **HDPPBA-2C** electrochemically oxidized by progressive increase of the oxidation potentials; b) TDDFT-B3LYP/6-31G**-calculated vertical transition energies.

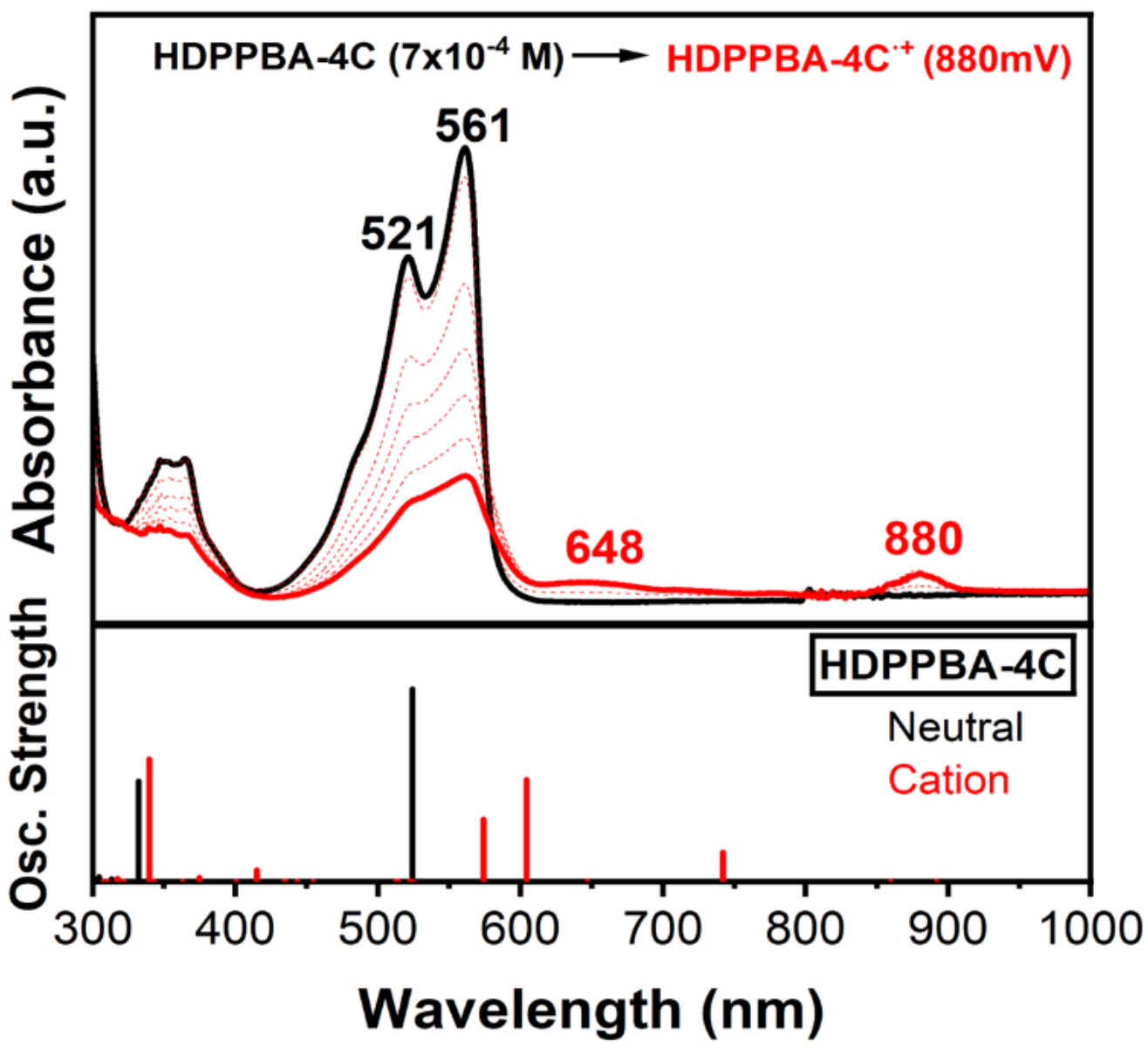

Figure S13. a) UV-Vis-NIR absorption spectra of **HDPPBA-4C** electrochemically oxidized by progressive increase of the oxidation potentials; b) TDDFT-B3LYP/6-31G**-calculated vertical transition energies.

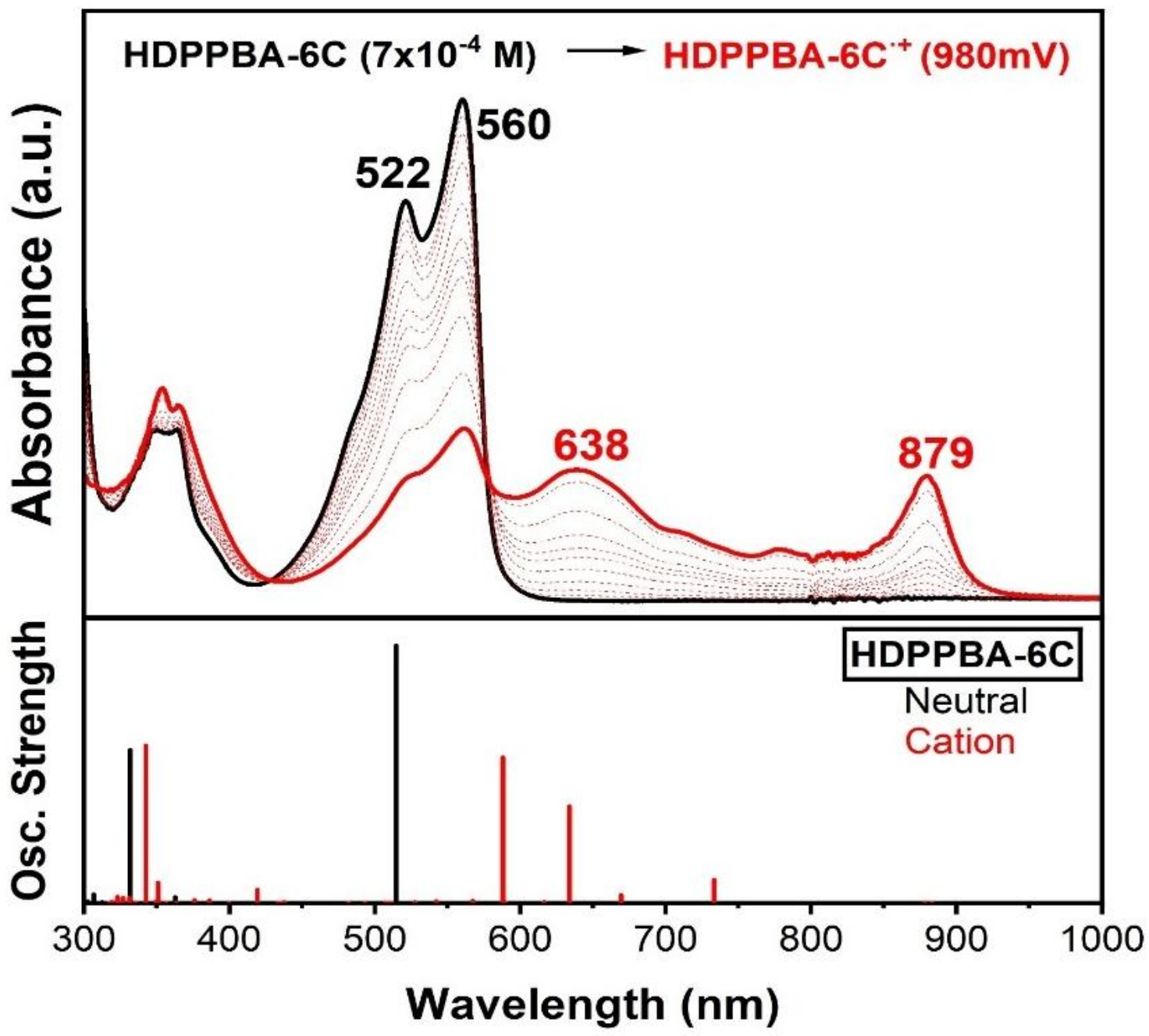

Figure S14. a) UV-Vis-NIR absorption spectra of **HDPPBA-6C** electrochemically oxidized by progressive increase of the oxidation potentials; b) TDDFT-B3LYP/6-31G**-calculated vertical transition energies.

Field effect transistors characterization

Organic field-effect transistors have been fabricated in a top-contact/bottom-gate structure. The device parameters, such as, the charged mobilities (μ), threshold voltage (V_T) and current on/off ratio (I_{ON} /I_{OFF}) were extracted from the saturated region in the device transfer curves using conventional transistors formalisms (eq (1))

$$I_{DS}=\frac{WC\mu}{2L}(V_G\text{-}V_T)^2 \qquad (1)$$

Where I_{DS} correspond to the drain current in the saturation regime, W to the channel width, L to the channel length, C is the capacitance per unit area of the insulator layer, and V_G the gate voltage.

The deposition of the active semiconducting film for the three compounds (**HDPPH**, **HDPPBA-2C, HDPPBA-4C** and **HDPPBA-6C**) has been carried out by drop casting from different solutions with a concentration of 3 mg/ml in chloroform at room temperature on a gate dielectric (d-doped Si wafers

with 300 nm thermally grown SiO_2 dielectric layer) previously functionalized with hexamethyldisilazane (HMDS) and octadecyltrichlorosilane (OTS) self-assembled monolayers (Table S3). The capacitance of the 300 nm SiO_2 gate insulator was 10 $nFcm^{-2}$. Prior the surface functionalization, the wafers were solvent cleaned by immersing them twice for 10 min in EtOH with sonication, drying with a stream N_2, and treating with UV-ozone for 10 min. For HMDS treatment, the cleaned silicon wafers were exposed to HMDS vapor at room temperature in closed air-free container under argon for a week, and for OTS treatment, the cleaned silicon wafers were immersed in OTS solution (3 mM) during 2 hours, after, the wafers were cleaned with hexane, acetone and EtOH respectively for 10 min with sonication and drying with a stream N_2. Subsequently of the deposition of active semiconducting films, OFETs devices were completed by gold vapor deposition through a shadow mask to define devices with various channel lengths and channel widths. Prior the electrode deposition, some of the substrates were subjected to a heat treatment (120 ºC during 3 hours), in order to modify the semiconducting thin film morphology and long-range order and thus improve the OFET performance parameters.

The devices were characterized under vacuum (in a customized probe station) with a 4200-SCS/C Keithley semiconductor characterization system.

The film layers were characterized by AFM (Figure S1) and GIXRD (Figure S2) measurements. AFM images were recorded by Multimode atomic force microscope with a Nanoscope V controller (Bruker Corporation, Billerica, MA, USA) working in tapping mode.

GIXRD data using CuKα1 radiation was recorded by using a Bruker D8 DISCOVER diffractometer. The grazing incidence X-ray diffraction setup is equipped with a parabolic Göbel mirror and a conventional line focus Cu radiation tube (40 kV/40mA).

Table S3. p-channel OFET derived electrical data for **HDPPH**, **HDPPBA-2C**, **HDPPBA-4C** and **HDPPBA-6C**. The best values and average (in parenthesis) are shown. The average values were obtained from at least 5 devices for each material.

Compound	Subst. Treatment	μ_h (cm^2 V^{-1} s^{-1})	V_T(V)	I_{ON}/I_{OFF}
**HDPPBA-2C**	OTS,(RT)	$8x10^{-6}$ ($5x10^{-6}$)	7	$7x10^{+0}$
**HDPPBA-2C**	OTS,(RT,120ºC)	$4x10^{-5}$ ($3x10^{-5}$)	-10	$4x10^{+1}$
**HDPPBA-2C**	HMDS,(RT)	$6x10^{-6}$ ($5x10^{-6}$)	30	$5x10^{+0}$
**HDPPBA-2C**	HMDS,(RT,120ºC)	$2x10^{-5}$ ($2x10^{-5}$)	-10	$1x10^{+1}$
**HDPPBA-2C**	ST,(RT)	NA	NA	NA
**HDPPBA-2C**	ST,(RT, 120ºC)	NA	NA	NA
**HDPPBA-4C**	OTS,(RT)	$3x10^{-3}$ ($2x10^{-3}$)	4	$1x10^{+4}$
**HDPPBA-4C**	OTS,(RT,120ºC)	$5x10^{-3}$ ($3x10^{-3}$)	-15	$3x10^{+3}$
**HDPPBA-4C**	HMDS,(RT)	$6x10^{-3}$ ($4x10^{-3}$)	-28	$2x10^{+5}$
**HDPPBA-4C**	HMDS,(RT,120ºC)	$6x10^{-3}$ ($6x10^{-3}$)	-5	$1x10^{+4}$
**HDPPBA-4C**	ST,(RT)	$2x10^{-4}$ ($1x10^{-4}$)	-22	$4x10^{+2}$
**HDPPBA-4C**	ST,(RT, 120ºC)	$7x10^{-4}$ ($5x10^{-4}$)	-22	$1x10^{+3}$
**HDPPBA-6C**	OTS,(RT)	NA	NA	NA
**HDPPBA-6C**	OTS,(RT,120ºC)	$2x10^{-2}$ ($2x10^{-2}$)	-12	$2x10^{+4}$
**HDPPBA-6C**	HMDS,(RT)	$6x10^{-3}$ ($5x10^{-3}$)	-11	$1x10^{+4}$
**HDPPBA-6C**	HMDS,(RT,120ºC)	$5x10^{-3}$ ($5x10^{-3}$)	-18	$5x10^{+3}$
**HDPPBA-6C**	ST,(RT)	$1x10^{-5}$ ($1x10^{-5}$)	-9	$2x10^{+1}$
**HDPPBA-6C**	ST,(RT, 120ºC)	$1x10^{-4}$ ($1x10^{-4}$)	-17	$1x10^{+2}$

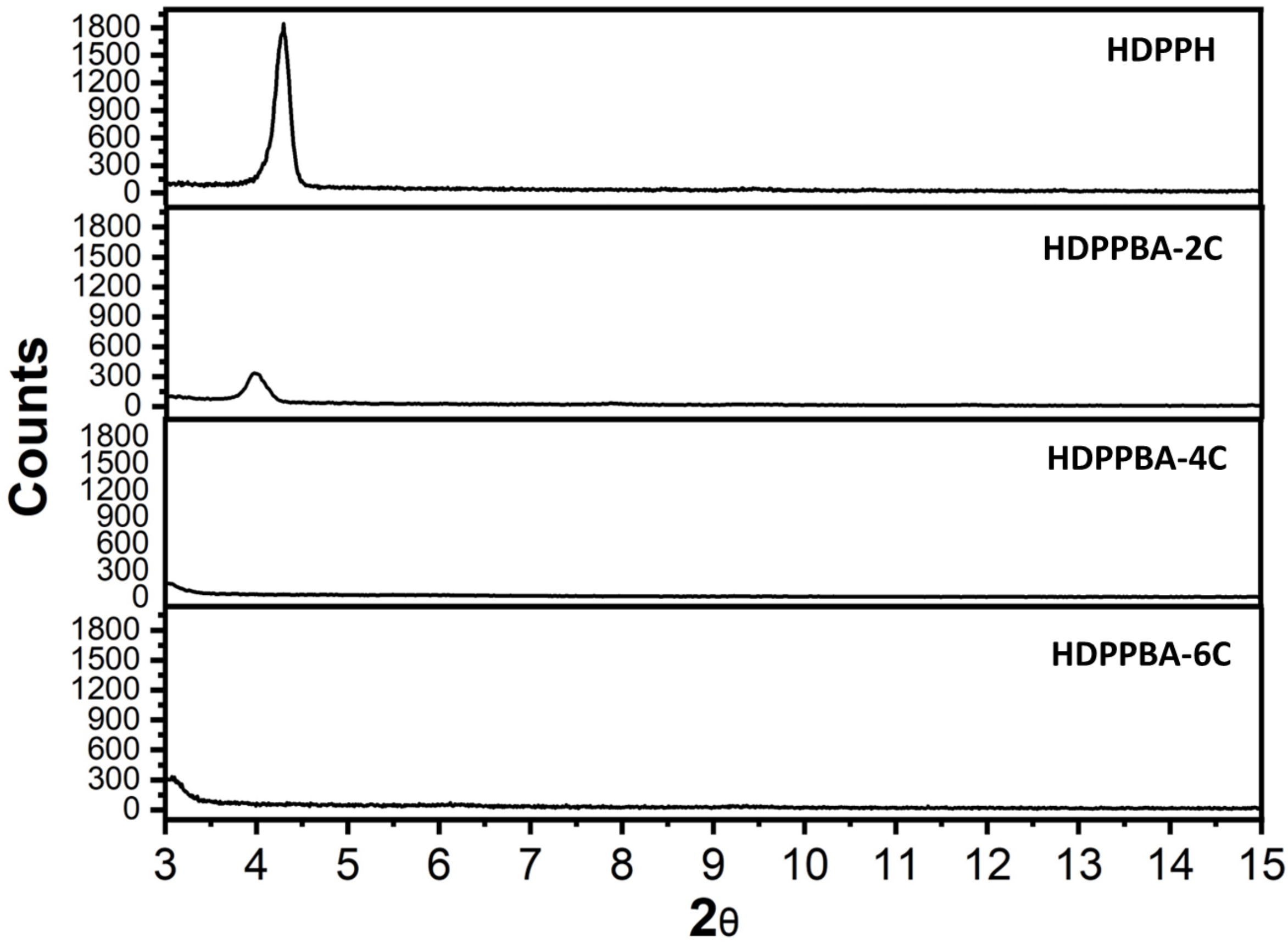

Figure S15. ϴ-2ϴ X-ray diffraction scans of solution deposited **HDPPH**, **HDPPBA-2C**, **HDPPBA-4C** and **HDPPBA-6C** thin films grown on OTS-treated Si/SiO_2 substrates and thermal annealing of 120ºC during 3 hours.

References

1 C. Lee, W. Yang and R. G. Parr, Development of the Colle-Salvetti correlation-energy formula into a functional of the electron density, *Phys Rev B*, 1988, 37, 785.

2 A. D. Becke, Density-functional thermochemistry. III. The role of exact exchange, *J Chem Phys*, 1993, 98, 5648–5652.

3 W. J. Hehre, K. Ditchfield and J. A. Pople, Self—Consistent Molecular Orbital Methods. XII. Further Extensions of Gaussian—Type Basis Sets for Use in Molecular Orbital Studies of Organic Molecules, *J Chem Phys*, 1972, 56, 2257–2261.

4 M. M. Francl, W. J. Pietro, W. J. Hehre, J. S. Binkley, M. S. Gordon, D. J. DeFrees and J. A. Pople, Self-consistent molecular orbital methods. XXIII. A polarization-type basis set for second-row elements, *J Chem Phys*, 1982, 77, 3654–3665.

5 E. Runge and E. K. U. Gross, Density-Functional Theory for Time-Dependent Systems, *Phys Rev Lett*, 1984, 52, 997.

6 H. H. Heinze, A. Görling, N. Rösch, J. Chem Phys, A. Gö rling and N. Rö sch, An efficient method for calculating molecular excitation energies by time-dependent density-functional theory, *J Chem Phys*, 2000, 113, 2088–2099.
